# Multi-omics analysis of saccharomyces boulardii supplementation reveals coordinated microbiome, metabolic, and immune signaling changes accompanying tumor suppression

**DOI:** 10.1080/19490976.2026.2690687

**Published:** 2026-06-30

**Authors:** Troels Holger Vaaben, Ditte Olsen Lützhøft, Karl Alex Hedin, Linda Ahonen, Ruben Vazquez-Uribe, Morten Otto Alexander Sommer

**Affiliations:** a Novo Nordisk Foundation Center for Biosustainability, Technical University of Denmark, Kgs. Lyngby, Denmark; b Center for Microbiology, Vlaams Instituut voor Biotechnologie, Leuven, Belgium

**Keywords:** Aryl Hydrocarbon Receptor, AhR, probiotic, multiomics, metagenomics, colorectal cancer

## Abstract

The gut microbiome shapes cancer progression and treatment responses, yet scalable microbiome-targeted interventions remain limited. We screened commercial probiotics for activation of the host aryl hydrocarbon receptor (AhR) and identified the yeast *Saccharomyces boulardii* as a consistent AhR activator. In an immunocompetent syngeneic colorectal cancer model, daily oral gavage of *S. boulardii* slowed growth of established subcutaneous tumors without detectable tumor colonization. Integrated profiling of the gut microbiome, circulating metabolites, cytokines, and tumor transcriptomes revealed a coordinated systemic response. *S. boulardii* increased microbial diversity and functionally rebalanced the gut microbiota, enriching taxa with lower genome-encoded biosynthetic autonomy. These changes were accompanied by elevated plasma levels of several indole metabolites, including the AhR agonists 5-hydroxyindole-3-acetic acid (5-HIAA) and indole-3-propionic acid (IPA). Targeted LC–MS/MS showed that *S. boulardii* can produce 5-HIAA under culture conditions, whereas IPA was not detected, suggesting that increased plasma levels of these metabolites may arise through a combination of probiotic activity and broader microbiome-associated processes. Circulating IL-17A and CTLA-4 were reduced, and tumors exhibited downregulation of programs linked to invasion, inflammation, and KRAS signaling. Multi-omics integration showed strong covariation across microbial, metabolic, immune signaling, and tumor compartments, highlighting coordinated cross-compartment responses during S. boulardii–associated tumor suppression.

## Introduction

The oral and gastrointestinal microbiomes have emerged as modulators of cancer progression and therapeutic outcomes. Growing evidence indicates that commensal microbes can shape systemic immunity and influence responses to anticancer treatments​.^1^ Melanoma patients who respond to anti–PD-1 immune checkpoint therapy have greater gut bacterial diversity and distinct microbial compositions compared to non-responders​.^2^ These favorable microbiota profiles correlate with enhanced anti-tumor immune activity. Moreover, fecal microbiota transfer from responder patients can confer improved tumor control in preclinical models​.^2^ Conversely, disruption of the microbiome by antibiotics has been associated with impaired efficacy of checkpoint inhibitors, supporting that the microbiome can modulate cancer immunosurveillance​.^1^ Taken together, these findings suggest that manipulating the gut microbiome can impact cancer therapy outcomes.

One mechanism linking the microbiome to tumor immunity is through microbiota-derived metabolites acting on host immune receptors. Notably, the aryl hydrocarbon receptor (AhR) is a ligand-activated transcription factor that senses small molecules from diet, commensal microbes, and host metabolism, thereby integrating environmental cues into immune responses​.^3^ Tryptophan metabolites produced by gut bacteria are prominent AhR ligands; for instance, an indole derivative from lactobacilli, indole-3-aldehyde, engages AhR to induce IL-22, reinforcing mucosal immune defenses​.^4^ Microbial indole derivatives not only enhance mucosal defenses but also modulate tumor immunity by fine-tuning the balance between pro‐ and anti‐inflammatory signals in the tumor microenvironment.^5^ In this context, indole-3-lactic acid (ILA), a tryptophan catabolite produced by *Lactobacillus reuteri*, has been shown to suppress colorectal tumorigenesis by downregulating IL-17A signaling pathways, a key driver of tumor-promoting inflammation. In preclinical models, ILA administration shows a significant reduction in tumor burden, highlighting its potential to re-establish immune homeostasis and inhibit cancer progression.^6^ Likewise, we recently demonstrated that another indole metabolite, indole-3-acetic acid (IAA), reprograms the tumor microenvironment via AhR, elevating CXCL9 and IFN-*γ*, increasing tumor-infiltrating T cells, and suppressing tumor growth.^7^

These insights have prompted efforts to modulate the gut microbiome as a novel approach in cancer treatment. Strategies under investigation include probiotic supplementation, treatment with advanced microbiome therapeutics,^7-9^ prebiotics, and fecal microbiota transplantation (FMT) aimed at enriching beneficial commensals​.^10^ Early clinical trials in advanced melanoma provide proof-of-concept, showing that FMT from immunotherapy-responsive donors into PD-1–refractory patients resulted in restored clinical responses in a subset of patients​.^11^ ^,^ ^12^

In parallel, certain probiotics like *Bifidobacterium BB-12* and *Lactobacillus rhamnosus* are being explored to mitigate treatment-related gastrointestinal toxicity^13^ while other species such as *Clostridium butyricum* CBM588 are under investigation for their ability to bolster anti-cancer immune responses^14^ thereby serving as adjuvants to existing cancer therapies. Notably, microbiome interventions are already applied to reduce antibiotic-associated *Clostridioides difficile* infection, illustrating the tangible benefits of microbiota modulation​.^15^ Although evidence for probiotic use in cancer remains preliminary, emerging data suggest that well-characterized strains can modulate the gut microbiota and systemic immunity to complement cancer therapies,^14^ ^,^ ^16^ ^,^ ^17^ or reduce treatment-induced side effects.^18-21^

Translating microbiome-based therapies into routine oncology practice requires rigorous validation. While FMT has shown promise in restoring responses to cancer immunotherapy, it presents notable challenges. The complexity of transferring an entire, minimally defined microbial community introduces issues related to standardization, donor variability, and long-term safety. Additionally, practical and regulatory hurdles, such as donor screening, preparation, and storage, hamper its scalability and widespread clinical application.^22^ In contrast, off-the-shelf probiotics offer a safe, practical and scalable alternative. Comprising well-defined microbial strains, these products are easier to manufacture and already possess established safety records.^23^ The widespread availability of over-the-counter probiotics provides a strong foundation for therapeutic exploration. While the safety of probiotics is often well-documented, their functional effects in both health and disease contexts, including cancer, remain less understood. Identifying which existing probiotics may exert beneficial effects represents an important next step. Leveraging the safety and accessibility of existing products could expedite identification of the most functionally relevant probiotics for specific applications, thereby streamlining their translation into clinical practice. In one study, 28.5% of patients diagnosed with cancer reported active use of probiotics,^24^ indicating that their use is already widespread despite limited understanding of whether commonly consumed strains have any measurable biological effects.

In this study, we screened commercially available probiotics for their ability to activate the AhR, aiming to evaluate their potential to modulate colorectal tumorigenesis as a step toward accessible, clinically viable microbiome-targeted interventions. Our screening revealed that probiotic products containing *Saccharomyces boulardii*, either as a single strain or within a multi-strain community, strongly activated AhR. In a mouse model of colorectal cancer (CRC) integrated with multi-omics phenotyping, oral *S. boulardii* supplementation significantly attenuated tumor growth, modulated the gut microbiome, and altered the systemic plasma metabolome and immune profile.

## Results

### Probiotic yeast *S. boulardii* activates AhR

**Figure 1.**
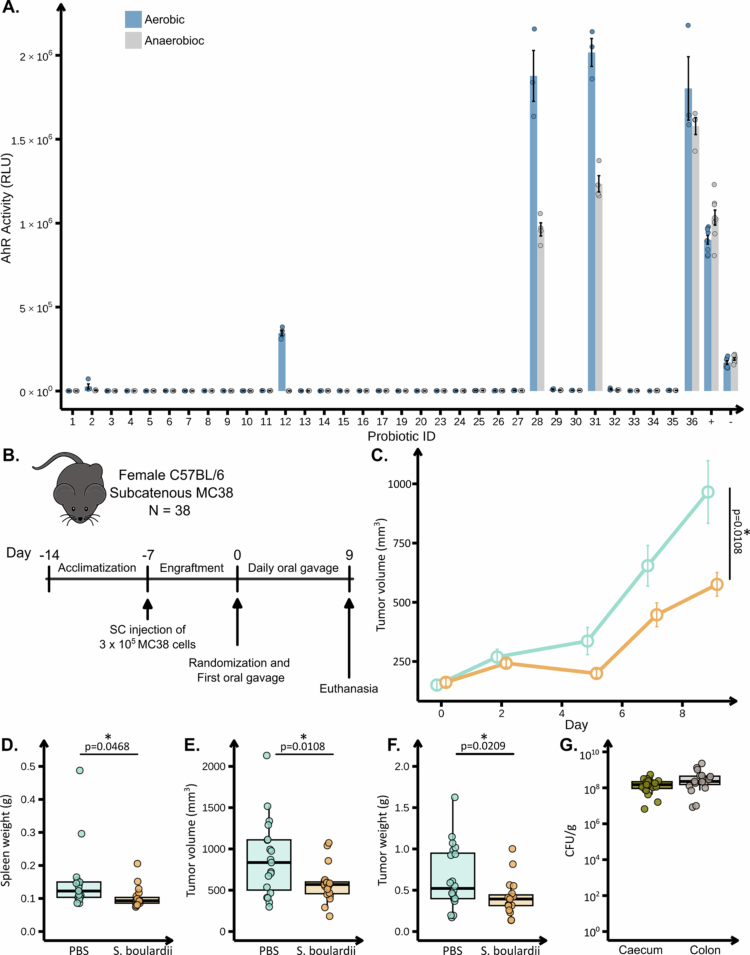
*In vitro* screening for AhR activity and *in vivo* evaluation of S. boulardii in a murine CRC model. (A) AhR activity of probiotic products available in Danish pharmacies. Activity was measured using a luciferase-based AhR reporter assay. Reporter cells were stimulated with 10% spent medium from probiotics grown once in MRS under aerobic or anaerobic conditions. Relative luminescence units (RLU) indicate AhR activation. FICZ (10 µM) served as the positive control; uninoculated MRS medium was the negative control. *n* = 4 biological replicates for probiotics, *n* = 8 for positive and negative controls. (B) Schematic of the MC38 colorectal cancer model. Seven-week-old female C57BL/6 mice were subcutaneously implanted with MC38 tumor cells on the right flank. Once tumors reached an average volume of 150 mm³, mice were randomized to receive daily oral gavage of either S. boulardii DBVPG 6763 (10⁸ CFU) or PBS for 9 days. All mice were euthanized after this period according to humane endpoints. (C) Tumor volume trajectory during the treatment period. (D) Spleen weight at endpoint. (E) Tumor volume at the final timepoint. (F) Tumor weight post-mortem. (G) Colony-forming units (CFU) of S. boulardii recovered from the cecum and colon content. Data are presented as mean ± SEM. Statistical significance was determined using two-sided Welch’s t-test or one-way ANOVA followed by Tukey’s post hoc test in cases of multiple comparisons (*n* = 20 for PBS; *n* = 19 for S. boulardii, *p < 0.05).

Given the established role of AhR activation in modulating immune responses and tumor progression, we first screened a panel of commercially available probiotic products (Supplementary Table 1) for their ability to activate AhR. Using a mammalian cell-based AhR reporter assay, each probiotic was tested under both aerobic and anaerobic conditions to reflect the gut environment and account for oxygen-sensitive metabolic activity (Figure 1A). Four products demonstrated notable AhR activation, with probiotic isolate ID 12 showing activity only in the presence of oxygen. Three products (IDs 28, 31, and 36) exhibited high AhR activity under both aerobic and anaerobic conditions, and all contained the probiotic yeast *S. boulardii*. Notably, *S. boulardii* was present both as a single strain formulation and as part of multi-strain probiotic communities. Furthermore, the different probiotic products contained distinct *S. boulardii* strains. This suggests that the capacity of *S. boulardii* to activate AhR is consistent across different formulations and strains. From probiotic ID 36, which demonstrated the strongest AhR activation under anaerobic conditions, we isolated *S. boulardii* DBVPG 6763. Given our prior findings^25^ and broader evidence supporting the role of AhR activation in cancer progression and immune modulation, this strain was selected for further testing to evaluate its potential to attenuate CRC growth in murine models.

### *S. boulardii* is a robust AhR activator and significantly slows tumor growth in murine CRC model

To assess the impact of *S. boulardii* on CRC progression, we employed an immunocompetent MC38 mouse model and allowed subcutaneous tumors to engraft prior to intervention. This model enabled investigation of systemic immune interactions and distal effects of probiotic supplementation. Tumors were established subcutaneously on the flank and once engrafted, mice were randomized based on tumor-size and bodyweight to daily oral gavage of 10^8^ CFU *S. boulardii* or PBS (Figure 1B). During the 9-day treatment, *S. boulardii* supplementation significantly slowed tumor growth relative to controls (Figure 1C). Post-mortem measurements confirmed this effect, as *S. boulardii*–treated mice showed lower spleen weight, tumor volume, and tumor weight (Figure 1D–F). Quantification of gut contents confirmed viable *S. boulardii* in both the cecum and colon (Figure 1G). Because the final gavage was given on the day of euthanasia, colonization could not be assessed, but *S. boulardii* was present in both cecum and colon content at ~10^8^ CFU/g at the time of euthanasia. Serial dilutions of tumor homogenates yielded no CFUs, indicating no detectable translocation to the subcutaneous tumors. Accordingly, oral supplementation with *S. boulardii* attenuated tumor growth at a distant site, suggesting a systemic mode of action.

### *S. boulardii* reshapes the plasma metabolome and elevates AhR agonists

**Figure 2.**
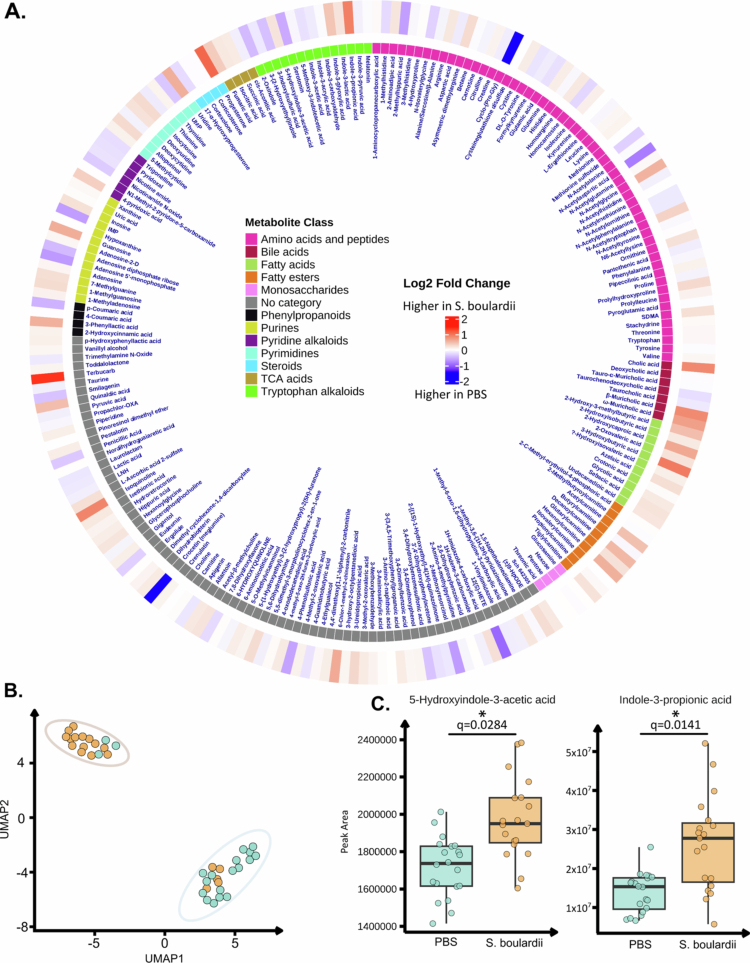
Plasma metabolomics reveals elevated AhR agonists following S. boulardii supplementation. (A) Untargeted metabolomics on plasma from mice treated with *S. boulardii* or PBS, shown as log_2_ fold-change (*S. boulardii* vs PBS) across 221 high-confidence metabolites, color-coded by chemical class. (B) UMAP projection of plasma metabolomics, reduced to two dimensions (n_neighbors = 10, min_dist = 0.3), with clusters defined by k-means. (C) Boxplots showing the levels of two AhR agonists, 5-hydroxyindole-3-acetic acid and indole-3-propionic acid, significantly elevated in the S. boulardii group. Boxes indicate median and interquartile range. For each metabolite, group differences were tested with a two-sided Welch’s t-test. Multiple comparisons across the 221 annotated metabolites were controlled with Benjamini–Hochberg false discovery rate; q-values are reported in panel C (*n* = 20 for PBS; *n* = 19 for S. boulardii, *q < 0.05)

To explore whether *S. boulardii* influenced systemic metabolic profiles, we next performed untargeted metabolomics on plasma samples. High-resolution untargeted LC–MS/MS of plasma detected 870 features: 221 were assigned high-confidence identifications (accurate mass with MS/MS and/or retention time confirmed against standards), 198 were putatively identified on accurate-mass/elemental-composition library matches, and 451 remained unannotated. For downstream analyzes, we focused quantitative comparisons on the 221 high-confidence metabolites. Global differences were modest in magnitude (log_2_ fold-change ≈ −2 to 2), but the overall metabolic profiles differed markedly between S. boulardii– and PBS-treated animals. Several chemical classes showed concordant shifts. For example, fatty acids and fatty esters were consistently elevated in *S. boulardii* relative to PBS treated animals (Figure 2A). UMAP analysis showed clear separation between *S. boulardii*–treated and control samples (Figure 2B), indicating substantial systemic metabolic modulation by *S. boulardii* treatment. After false discovery rate (FDR) correction, 16 metabolites differed significantly between the *S. boulardii* and control group (Supplementary Figure S1).

Among the significantly altered metabolites, 5-hydroxyindole-3-acetic acid (5-HIAA) and indole-3-propionic acid (IPA) were elevated in the *S. boulardii* group (log_2_ fold-change: +0.20 and +0.92, respectively; Figure 2C) both of which have been described as AhR agonists.^26^ IPA is predominantly produced by obligate anaerobic gut bacteria (e.g., *Clostridium sporogenes*) and has not been reported in *S. boulardii* or *S. cerevisiae*, raising the question of whether the increased plasma IPA could originate directly from the probiotic. To evaluate this, we performed targeted LC–MS/MS analysis of spent *S. boulardii* culture supernatants under aerobic and anaerobic conditions (Figure S2A). 5-HIAA was detectable in both conditions and was substantially higher than in sterile MRS controls, with markedly elevated levels under anaerobic growth. These observations are consistent with prior findings in *S. cerevisiae*, where 5-HIAA production increases under acidic stress conditions.^27^ In contrast, IPA was not detected in any *S. boulardii* culture despite clear chromatographic detection in calibration standards (Figure S2B). Together, these data indicate that S. boulardii could contribute to circulating AhR-active metabolites through production of 5-HIAA, while IPA was not detected in S. boulardii culture supernatants. The elevated plasma IPA observed *in vivo* is therefore consistent with the hypothesis of an indirect, microbiome-mediated effect, suggesting that increased IPA originates from community-level changes rather than direct yeast metabolism.

### *S. boulardii* modulates the microbiota composition and increases alpha diversity

We previously showed that *S. boulardii* significantly reshapes microbial communities,^25^ for example, by supplying amino acids that support the growth of other microbes. We hypothesized that the elevated plasma IPA was not produced directly by *S. boulardii* but was an indirect effect of microbiome modulation. We performed metagenomic sequencing on colonic contents after treatment to determine how *S. boulardii* altered the microbiome, potentially influencing IPA production and other systemic metabolic changes.

**Figure 3.**
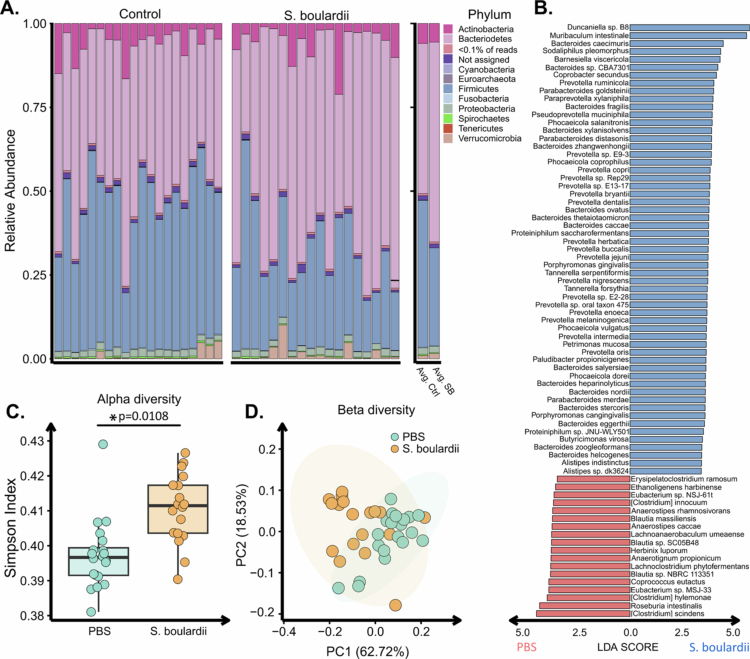
*S. boulardii* supplementation reshapes the gut microbiome in tumor-bearing mice. (A) Phylum-level relative abundance profiles from metagenomic sequencing of colon content, grouped by treatment. Each stacked bar represents one mouse; additional bars display the average profile for the PBS and *S. boulardii* groups. (B) Differentially abundant microbial species identified with LEfSe, displayed as LDA scores indicating enrichment in PBS or *S. boulardii*–treated groups; group differences were tested by two-sided Kruskal–Wallis and Wilcoxon rank-sum tests with Benjamini–Hochberg FDR control (q < 0.05), and taxa with LDA > 2.0 were reported as enriched. (C) Alpha diversity assessed by Simpson’s diversity index (1—D); group differences were evaluated using two-sided Welch’s t-test. (D) Beta diversity assessed by Principal Coordinates Analysis (PCoA) of Bray–Curtis dissimilarity, with statistical differences evaluated using PERMANOVA (999 permutations). Cluster centroids and convex hulls are overlaid (*n* = 20 for PBS; *n* = 18 for S. boulardii, *p < 0.05)

Taxonomic profiling revealed notable shifts with *S. boulardii* treatment increasing Bacteroidetes and reducing Firmicutes (Figure 3A). An elevated Firmicutes/Bacteroidetes ratio is consistently associated with CRC, with higher ratios in CRC patients and individuals with polyps relative to healthy controls.^28^ Moreover, in cancer patients, increased Firmicutes abundance is associated with poor therapeutic response.^29^ To pinpoint taxa driving this shift, we performed Linear discriminant analysis Effect Size^30^ (LEfSe), which identified several altered species (Figure 3B). Multiple *Prevotella* species, a genus linked to favorable CRC prognosis^31^, were significantly increased in the *S. boulardii* group. Similarly, we observed an enrichment of *Muribaculum intestinale* in the *S. boulardii* group. *M. intestinale* modulates host immunity by shaping T-cell populations via microbial metabolites.^32^ It also produces 3-hydroxybutyric acid,^33^ which was significantly elevated in the plasma of *S. boulardii*-treated mice (Supplementary Figure S1), consistent with its increased abundance and supporting a link between probiotic-driven microbiome shifts and systemic metabolic effects. Conversely, *Clostridium scindens*, a bacterium implicated in CRC pathogenesis via secondary bile acid metabolism,^34^ was markedly reduced by *S. boulardii* treatment. Colonization by *C. scindens* and related bile acid–metabolizing bacteria increases CRC risk and severity in animal models,^35^ highlighting a potential beneficial mechanism of *S. boulardii* treatment.

To evaluate probiotic-induced changes in community structure, we examined both alpha- and beta-diversity. *S. boulardii* supplementation significantly increased alpha diversity, indicating greater microbial richness and evenness (Simpson’s diversity index; Figure 3C). Likewise, principal coordinates analysis (PCoA) of Bray–Curtis distances showed significant separation in microbiome composition between *S. boulardii* and control mice (PERMANOVA on Bray–Curtis, R² = 0.201, *p* = 0.001; Figure 3D). Thus, *S. boulardii* treatment reshapes the gut microbiome, promoting a more diverse and distinct community composition, a feature associated with improved CRC outcomes.^36^

To test the consistency of probiotic-induced microbiome shifts (including non-linear patterns), we trained an extreme gradient boosting (XGBoost) classifier to distinguish treatment groups. We trained the model on microbial relative abundance profiles (taxa as features) from *S. boulardii–*treated and PBS mice (see Methods for parameters). The XGBoost classifier accurately separated probiotic-treated from control mice, achieving a cross-validated ROC-AUC of 0.889 (Supplementary Figure S3A + S3C). Many top features from the model overlapped with LEfSe-identified species, underscoring their robust association with *S. boulardii* treatment (Supplementary Figure S3B). In addition, the classifier identified two important predictor species that LEfSe did not: *Akkermansia muciniphila* and *Ligilactobacillus murinus*. *A. muciniphila* is a gut commensal linked to improved cancer therapy responses, via enhanced intestinal integrity and reduced systemic inflammation,^37^ and was increased by *S. boulardii*, while *L. murinus* was decreased (Figure S3D). Collectively, the high accuracy of the model confirms that *S. boulardii* induced consistent microbiome changes, within the context of this experiment.

### *S. boulardii* reshapes microbiome function and promotes growth of Low Metabolic Independence species

**Figure 4.**
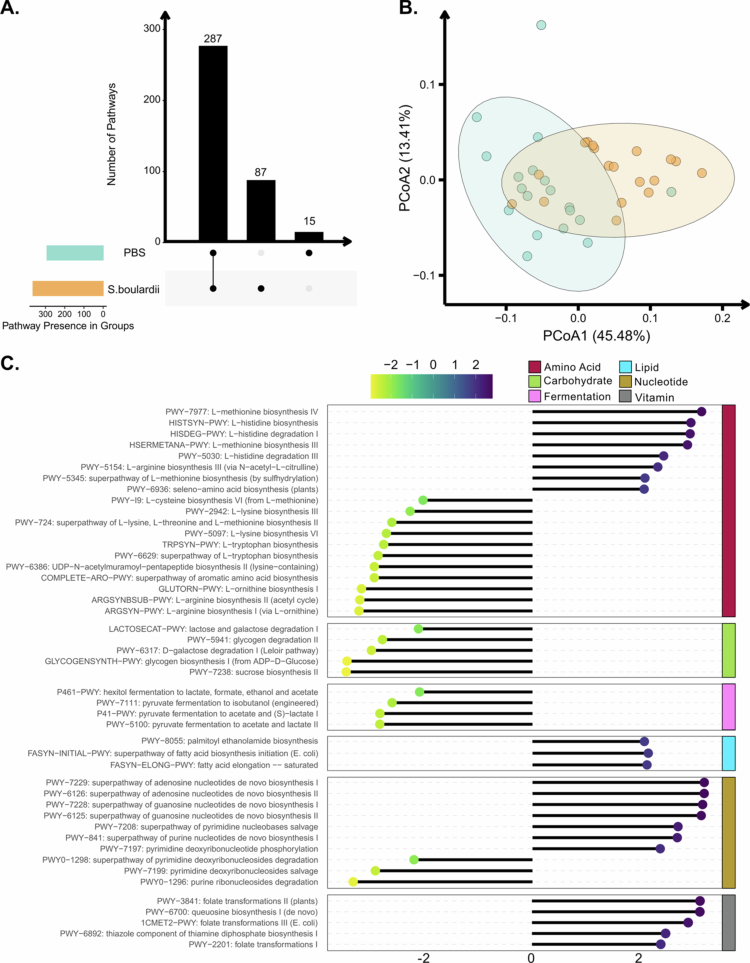
Functional restructuring of the gut microbiome following *S. boulardii* supplementation. (A) Pathway presence-absence matrix derived from HUMAnN3 MetaCyc annotations showing the number of pathways shared between or unique to treatment groups. (B) Principal Coordinates Analysis (PCoA) of Bray–Curtis dissimilarity based on pathway-level functional profiles reveals separation between groups. PERMANOVA with 999 permutations was used to assess statistical significance. (C) Differentially abundant microbial pathways from HUMAnN3 profiles identified with LEfSe, displayed as LDA scores indicating enrichment in PBS or *S. boulardii*–treated groups; group differences were tested by two-sided Kruskal–Wallis and Wilcoxon rank-sum tests with Benjamini–Hochberg FDR control (q < 0.05), and pathways with LDA > 2.0 were reported as enriched, colored by metabolic category (*n* = 20 for PBS; *n* = 18 for *S. boulardii*).

To connect compositional changes to functional shifts, we profiled microbial pathways using HUMAnN3 and stratified UniRef90 gene families by MetaCyc and Enzyme Commission (EC) annotations. Most metabolic pathways were shared between groups (287/389; Figure 4A); however, *S. boulardii*–treated mice harbored 87 unique pathways versus only 15 in controls. PCoA of pathway profiles confirmed a significant functional shift between groups (PERMANOVA on Bray–Curtis, R² = 0.205, *p* = 0.001; Figure 4B).

*S. boulardii* treatment broadly reprogrammed the functional gut microbiome. Vitamin metabolism pathways, including folate, queuosine, and thiamine biosynthesis were found in higher abundance in *S. boulardii* treated animals, while carbohydrate metabolism was broadly decreased, including reduced presence of glycolytic fermentation as well as glycogen and lactose degradation pathways (Figure 4C). This metabolic signature mirrors recent *ex vivo* co-culture findings in which *S. boulardii* similarly suppressed carbohydrate metabolism while enhancing nucleotide biosynthesis.^38^ Consistent with these pathway changes, 4 of the 16 differentially abundant plasma metabolites (25%) were nucleotide-related (e.g., uridine, guanosine, 7-methylguanine, isocytosine; Supplementary Figure S1), suggesting that increased microbial nucleotide production capacity was also reflected in the host circulation.

Amino acid biosynthesis pathways for lysine, phenylalanine, threonine and tryptophan were less abundant in *S. boulardii*–treated animals (Figure 4C). We previously demonstrated that *S. boulardii* donates amino acids in co-culture with bacteria^25^ and therefore posited that the reduced community-level amino acid biosynthesis reflects extracellular metabolite provisioning by *S. boulardii*. Recent genome-resolved analyzes show that stressed gut environments, including inflammatory bowel disease and CRC, are enriched for high-metabolic-independence (HMI) species, whose genomes encode complete de novo biosynthetic modules^39^; 33 HMI-associated KEGG modules that are hallmarks of species selected for in microbial communities in stressed gut environments have been identified,^40^ the majority of which are related to amino acid biosynthesis (Supplementary Table 2).

Motivated by the observed decrease in amino-acid biosynthesis and the concurrent rise in *α*-diversity from *S. boulardii* supplementation, we hypothesized that introducing *S. boulardii* would relax selection for HMI taxa and promote the growth of low metabolic independence (LMI) species in our murine cancer model. To test this hypothesis, we reconstructed metagenome-assembled genomes (MAGs) from colonic metagenomes and assigned each MAG a metabolic independence score summarizing genome-encoded completeness of amino acid, nucleotide, and vitamin/cofactor biosynthesis and central metabolism.

**Figure 5.**
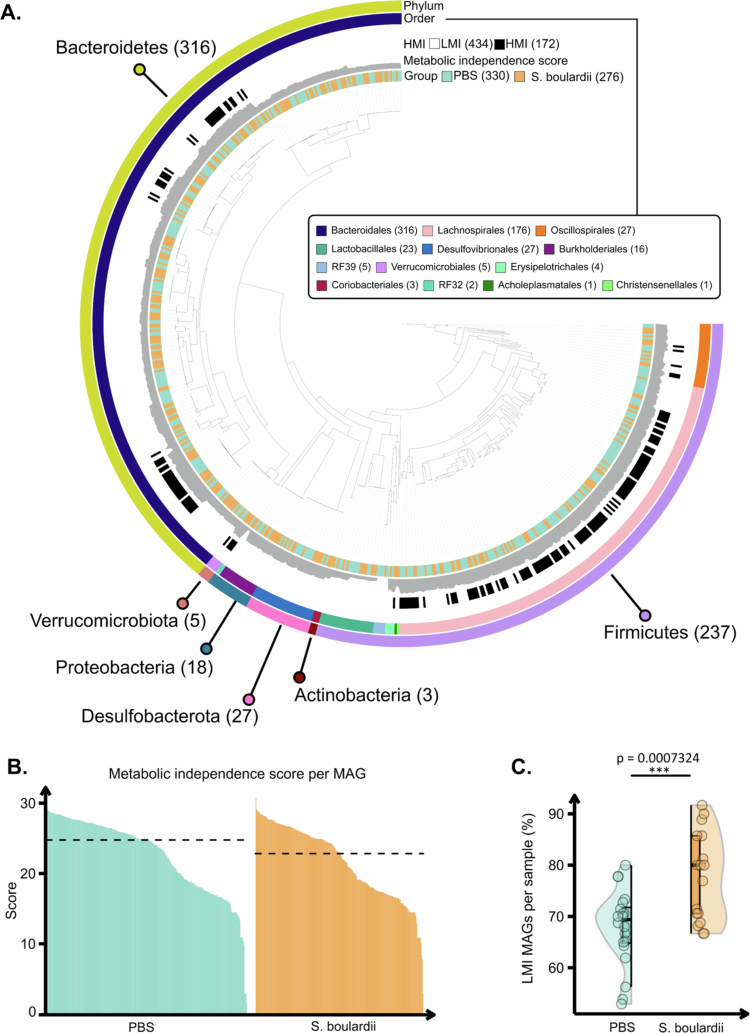
*S. boulardii* supports growth of low metabolic independence taxa. (A) Phylogeny of 606 MAGs retrieved from colonic metagenomes as assigned by Genome Taxonomy Database (GTDB) with concentric annotation rings. From the center outward: group of origin for each MAG (PBS vs *S. boulardii*), metabolic independence score, metabolic independence status (High vs Low), taxonomic order, and taxonomic phylum. (B) MAG metabolic independence scores by treatment group. Dotted line shows the median for each group. (C) Per-sample percentage of MAGs classified as low metabolic independence (LMI) (Two-sided Welch’s t-test, ***p < 0.001).

Across all samples 606 high-quality MAGs were reconstructed (Figure 5A; Supplementary Table 3), most assigned to Bacteroidetes or Firmicutes. Relative to PBS controls, MAGs from *S. boulardii*-treated animals had lower metabolic independence scores (Figure 5B) and a significantly higher per-sample fraction of LMI MAGs (68% in PBS versus 77% in *S. boulardii* group; Figure 5C; Two sided Welch’s t-test, *p* = 0.00073). HMI MAGs enriched in PBS were taxonomically concentrated within Firmicutes, particularly Lachnospirales. Thus, *S. boulardii* treatment reduced the prevalence of HMI lineages, most notably Firmicutes/Lachnospirales, shifting the genome-resolved community toward LMI taxa.

Our *in vitro* screen showed that *S. boulardii* activates AhR, and prior studies indicate that it both produces and stimulates the production of indole derivatives in co-culture.^25^ Consistent with this, we observed increased plasma levels of IPA and 5-HIAA, both known AhR agonists. Therefore, we examined microbial pathways involved in tryptophan metabolism to assess how *S. boulardii* influences indole-derivative production. We focused on key enzymatic steps converting tryptophan into a range of indole derivatives and quantified their representation across treatment groups (Supplementary Figure S4A). Four enzymatic steps showed significant increases in abundance in *S. boulardii–*treated mice, including those catalyzing the conversion of tryptophan to indole-3-pyruvate and further to indole-3-acetaldehyde. We assigned each enzyme’s abundance to contributing taxa using HUMAnN3’s species-stratified profiles, which map reads to sample-specific pangenomes of the detected species and then regroup gene families to EC steps. Species-level analysis revealed distinct contributors to these enzymatic shifts. The increase in enzyme catalyzing indole-3-pyruvate to indole-3-acetaldehyde (EC 4.1.1.74) was almost entirely mapped to *S. boulardii* (Supplementary Figure S4B), indicating a direct metabolic contribution. In contrast, increases in upstream enzymes (e.g., EC 2.6.1.1) were driven mainly by non-*S. boulardii* taxa, indicating that the expanded indole metabolic capacity was also the result of an indirect effect of community restructuring. Although the final enzymatic step in IPA production (EC 1.3.1.31) did not increase, the preceding reactions were consistently upregulated. This disconnect aligns with reports that IPA levels often rise *in vivo* without clear metagenomic signatures, reflecting gaps in current annotations and likely uncharacterized producers.^41^

### *S. boulardii* changes plasma cytokines to a profile associated with increased survival

**Figure 6.**
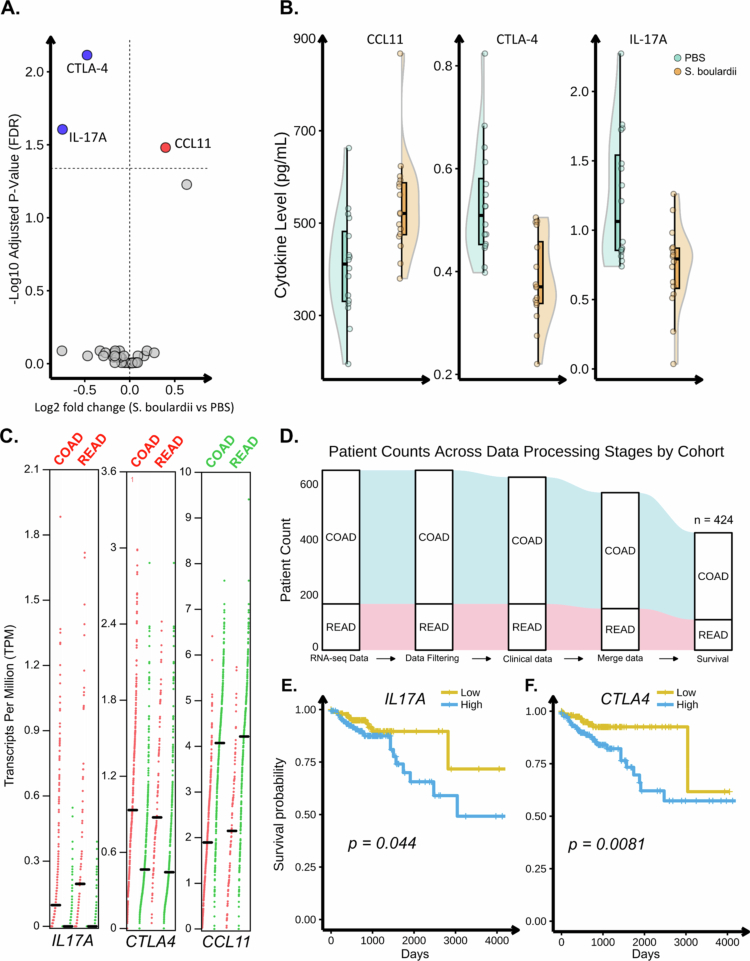
*S. boulardii* alters circulating cytokines to a profile associated with improved survival in colorectal cancer. (A) Volcano plot showing log₂ fold change (*S. boulardii* vs PBS) versus –log_10_ FDR-adjusted *p*-value for 43 plasma cytokines measured using the Olink Mouse Exploratory Panel. Group differences were tested with a two-sided Welch’s t-test, using Benjamini–Hochberg false discovery rate (*n* = 16 for PBS; *n* = 16 for *S. boulardii*)**.** (B) Raindrop plots of significantly different cytokines (CCL11, CTLA-4, IL-17A) showing distribution, median, and interquartile range. (C) Gene expression of IL-17A, CTLA-4, and CCL11 in colorectal tumor samples from the TCGA COAD and READ datasets, shown as transcripts per million (TPM); red labels indicate higher expression in tumor tissue, and green labels indicate higher expression in normal tissue. (D) Sample retention and cohort sizes across RNA-sequencing preprocessing and clinical integration steps in TCGA COAD and READ cohorts. (E, F) Kaplan–Meier survival curves of TCGA CRC patients stratified into high and low expression groups based on a median split of IL-17A (E) and CTLA-4 (F). Statistical significance was assessed using log-rank test.

Given evidence that *S. boulardii* modulates local mucosa^42^ and systemic immune responses^43^ in humans, we examined whether probiotic supplementation altered plasma cytokine levels in tumor-bearing mice. Using a targeted proteomics panel, we measured 43 plasma cytokines relevant to inflammation and cancer immunology. *S. boulardii*–treated mice had significantly lower circulating IL-17A and CTLA-4 levels and higher CCL11 levels (Figure 6A, B). Both IL-17A and CTLA-4 can drive tumor-promoting immunosuppression in CRC, with IL-17A promoting angiogenesis and chronic inflammation and CTLA-4 impairing T-cell effector function. Increased levels of IL-17A and CTLA-4 are correlated with poor immunotherapy response^44-46^ In contrast, CCL11, though context-dependent, has been linked to beneficial immune modulation and tumor control.^47^

Because murine and human immune systems differ in cytokine biology and tumor ecology, we contextualized these findings in human CRC by querying TCGA^48^ colon (COAD) and rectal (READ) expression profiles. IL-17A and CTLA-4 were significantly upregulated in CRC tumors compared to healthy colon (GTEx dataset^49^) whereas CCL11 was decreased (Figure 6C). These data indicate that *S. boulardii* supplementation partially reverses CRC-associated cytokine changes, shifting the systemic immune profile toward a profile closer to that of healthy individuals. To assess prognostic relevance, we pooled TCGA COAD/READ cases retained after RNA-sequencing and filtering for clinical metadata (*n* = 424; Figure 6D). For each gene, patients were stratified into low and high expression groups using a median split. Kaplan–Meier analyzes showed that the high-expression groups for IL-17A and CTLA-4 had significantly shorter overall survival than the low-expression groups (log-rank test *p* = 0.044 and *p* = 0.0081, respectively; Figure 6E–F). Thus, in humans, lower tumor expression of IL-17A and CTLA-4 associates with better outcomes. This matches the *S. boulardii*-treated mice, in which circulating IL-17A and CTLA-4 were reduced, linking the probiotic-induced cytokine shift to a pattern associated with improved prognosis.

### *S. boulardii* suppresses oncogenic and inflammatory gene programs in distant tumors

**Figure 7.**
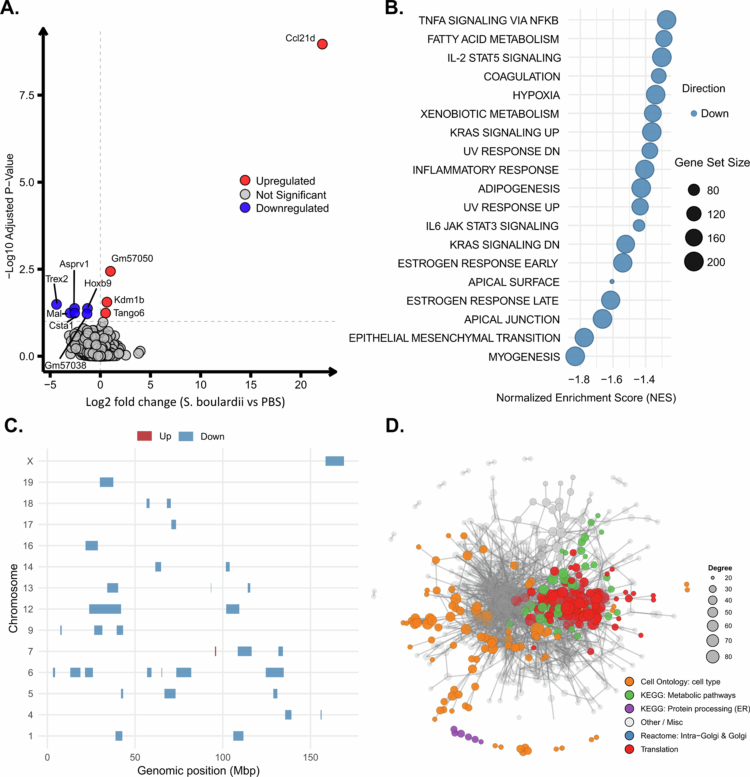
RNA-sequencing reveals transcriptomic alterations in tumors following *S. boulardii* treatment. (A) Volcano plot of differential gene expression from DESeq2 analysis comparing tumors from *S. boulardii*–treated and PBS control mice (*n* = 20 for PBS; *n* = 19 for *S. boulardii*). Genes with adjusted *p*-value < 0.1 (Benjamini–Hochberg correction) are shown as significantly upregulated (red) or downregulated (blue). (B) Gene Set Enrichment Analysis (GSEA) of Mouse Hallmark pathways using ranked gene lists. Normalized Enrichment Scores (NES) are shown for pathways significantly enriched at FDR < 0.1. Dot size indicates gene set size; all significantly enriched pathways in this dataset were negatively enriched. (C) Genomic regions identified using PREDA applied to DESeq2 Wald statistics. Each segment represents a significant genomic region (FDR < 0.1) along the mouse genome, with chromosome position shown on the x-axis and chromosome identity on the y-axis. Upregulated and downregulated regions are indicated by color. (D) *Protein–protein interaction network of genes located within PREDA-defined downregulated regions. Genes overlapping significant downregulated genomic intervals were mapped to STRING and used to construct an interaction network (STRING score ≥ 400), followed by Louvain community detection to identify densely connected modules. Nodes are colored by the most significantly enriched functional category per module (GO/KEGG/Reactome/UniProt keywords), and node size reflects degree (number of connections).*

Given the alterations in circulating metabolites and cytokines in the *S. boulardii* group relative to the control group, we examined the transcriptome of tumors from both groups. Bulk RNA-sequencing of endpoint tumor samples revealed that *S. boulardii* caused modest changes in gene expression profiles, with only 10 significantly differentially expressed genes (DEGs) (Figure 7A). Among the upregulated genes, Ccl21d exhibited the largest increase with a 22 log₂ fold-change. Ccl21d encodes a chemokine involved in the recruitment of dendritic cells and T cells, and its elevated expression may reflect a more immune-permissive tumor microenvironment.

Despite the low number of significant DEGs, gene set enrichment analysis (GSEA) revealed coordinated downregulation of multiple oncogenic and immunomodulatory programs in tumors from *S. boulardii*–treated mice (Figure 7B). Downregulated pathways included Hallmark gene sets for epithelial–mesenchymal transition, KRAS signaling, IL6–JAK–STAT3 signaling, inflammatory response, and IL2–STAT5 signaling. Notably, most genes were unique to individual enriched pathways rather than shared among multiple pathways (Figure S5A), indicating that the enrichment signal was not driven by a small set of overlapping genes but instead reflected broader pathway-level regulation. Because only 10 genes met the differential-expression threshold, these pathway results should be interpreted as coordinated transcriptomic shifts across ranked genes rather than validation of individual pathway drivers.

To investigate whether these modest gene-level changes were spatially coordinated along the genome, we applied PREDA to the DESeq2 Wald statistics. PREDA identified multiple significant genomic regions, predominantly showing downregulated smoothed statistics across chromosomes (Figure 7C), consistent with regional suppression of transcriptional activity rather than isolated single-gene effects.

We next examined the functional connectivity of genes located within PREDA-defined downregulated regions using STRING-based protein–protein interaction networks. Genes overlapping these regions formed densely connected subnetworks with distinct functional modules enriched for translation, metabolic pathways, protein processing in the endoplasmic reticulum, intra-Golgi and Golgi–ER trafficking, and cell-type–associated terms (Figure 7D).

Given the increase in circulating AhR agonists, we assessed whether tumors showed transcriptional evidence of AhR pathway activation. We quantified AhR pathway engagement by analyzing mean expression of a curated AhR target-gene set from the ChEA database (based on integrated ChIP-X experiments.^50^) The set contains 666 human AhR targets, of which 615 mapped to mouse gene identifiers. Of these, 581 genes with non-zero expression were included in the analysis. Mean expression of these AhR targets was significantly higher in tumors from *S. boulardii*–treated mice (Welch’s two-sample *t*-test, *p* = 0.0069; Figure S5B), indicating a modest but detectable transcriptional shift consistent with increased systemic exposure to AhR agonists. Because this ChEA-derived gene set integrates AhR targets identified across diverse cell types and experimental contexts, this analysis should be interpreted as a global transcriptional trend consistent with AhR pathway engagement, rather than evidence of direct AhR regulation within tumor cells, or proof that AhR is required for the anti-tumor effect.

To evaluate whether *S. boulardii* altered immune-cell composition within tumors, we applied mMCP-counter^51^ to infer immune and stromal populations from bulk RNA-seq profiles. No cell types showed statistically significant differences between treatment groups after FDR correction, and global composition analysis likewise did not reveal detectable shifts (Figure S5C). However, because bulk deconvolution provides only an indirect estimate of cellular abundance, these findings do not exclude the possibility of more subtle or spatially restricted immune changes. Definitive assessment of immune-cell composition would require orthogonal approaches such as flow cytometry or histological profiling, but within the resolution of our transcriptomic data, we did not detect robust differences in immune infiltration between groups.

We next asked whether the protein biomarkers altered in plasma (Figure 6A−B) might reflect transcriptional regulation within the tumor itself. We therefore examined tumor expression of *Ccl11*, *Ctla4*, and *Il17a* (Figure S5D). Ctla4 showed a concordant decrease at the transcript level, whereas *Il17a* and *Ccl11* did not differ between groups, suggesting that the reduced circulating IL-17A and elevated CCL11 likely originate from non-tumor sources. A plausible explanation for the drop in circulating IL-17A is reduced intestinal IL-17 production, as gut microbiota composition and microbiota-derived metabolites are known to tune mucosal Th17/γδ17 programs and IL-17 output *in vivo,* ^52^ ^,^ ^53^ thereby exerting measurable impact on circulating IL-17A levels. Collectively, these findings indicate that the plasma cytokine changes are only partly mirrored at the tumor transcript level, supporting a systemic immune effect that is not necessarily tumor-derived.

In summary, the tumor transcriptome displayed a subtle but coordinated pattern of regulation, characterized by broad regional downregulation of multiple tumor-promoting programs alongside a modest increase in AhR-responsive gene expression. These integrated changes suggest a coherent, system-level transcriptional response to treatment with *S. boulardii*.

### Integrated multi-omics reveals coordinated host–microbiome interactions

**Figure 8.**
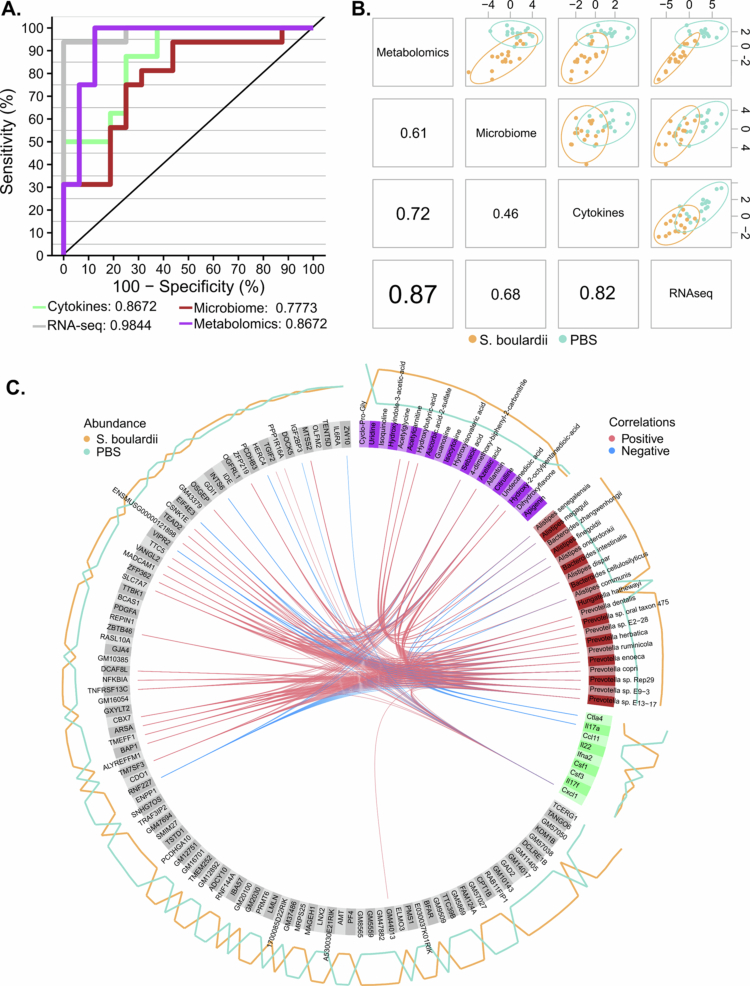
DIABLO integration highlights treatment-linked multi-omics signatures. (A) ROC curves for component 1 of each omics block. Cytokines (green), Microbiome (purple), Metabolomics (brown) and RNA-seq (gray) all display high discriminatory power; corresponding AUC values are indicated in the legend. (B) Matrix summarizing agreement between blocks on component 1. Upper-right panels show pairwise scatterplots of sample scores with 95% confidence ellipses (orange = *S. boulardii*, teal = PBS). Lower-left panels report Pearson correlation coefficients (r) between block scores, reflecting the strength of cross-omics concordance captured by DIABLO. (C) Circos plot depicting inter-block feature correlations (|r| ≥ 0.8) on component 1. Links are colored red (positive) or blue (negative). The outer ring traces group-specific abundance/expression for each feature (orange = *S. boulardii*, teal = PBS), enabling simultaneous visualization of differential regulation and multi-omics connectivity. Analysis restricted to animals with complete data across all omics blocks; *n* = 16 for PBS; *n* = 16 for *S. boulardii*.

To assess whether *S. boulardii* induced a coordinated shift across host and microbial compartments, we applied DIABLO,^54^ a multiblock sparse Partial Least Squares Discriminant Analysis (sPLS-DA) framework that integrates multiple omics datasets by maximizing inter-block covariance while maintaining class separation. Metabolomics, taxonomic microbiome data, cytokine levels, and tumor RNA-seq data were integrated under an equal-weight design matrix to extract shared latent components. This approach identifies features that are both discriminative of treatment groups and correlated across omics layers. Classification based on the first two latent components effectively separated *S. boulardii*–treated and PBS mice in all omics blocks (AUC range, 0.777–0.984; Figure 8A). Surprisingly, RNA-sequencing on tumor tissue achieved the highest classification performance (AUC = 0.984), suggesting that tumor-intrinsic transcriptional responses most sensitively captured the effect of treatment. Metabolomics and cytokine blocks also showed strong separation (AUC = 0.867), while microbiome profiles retained modest discriminative power despite higher inter-individual variability. Sample projections on component 1, i.e., the latent variables extractedfrom each omics block, were positively correlated across all pairwise combinations (Figure 8B). The strongest association was observed between the metabolomics and RNA-seq blocks (R² ≈ 0.87), indicating that the DIABLO integration successfully captured shared multivariate structure between circulating metabolites and tumor-intrinsic transcriptional programs. Lower, but still substantial, correlations were observed for microbiome and cytokine blocks (R² = 0.46–0.68), consistent with coordinated but partially distinct systemic responses.

To define cross-omics relationships among treatment-discriminative features, we quantified pairwise correlations between selected variables across all blocks (Figure 8C). The resulting network highlighted severalstrong correlations, particularly between microbial taxa, circulating cytokines, and tumor-intrinsic transcripts. Several *Prevotella* species formed densely connected hubs and were elevated in *S. boulardii*–treated mice. This genus has been associated with favorable prognosis in CRC patients, including lower risks of recurrence and disease-specific mortality after controlling for clinical covariates.^31^

These findings indicate that *S. boulardii* is associated with coordinated shifts across microbial, metabolic, immune, and tumor transcriptional compartments, consistent with coordinated systems-level responses accompanying reduced tumor progression.

## Discussion

Oral supplementation with *S. boulardii* reduced tumor growth in a subcutaneous CRC model alongside systemic changes in metabolism and immune signaling. While *S. boulardii* is commonly used or recommended for gastrointestinal indications, including prevention of antibiotic-associated diarrhea^55^ and adjunctive therapy alongside antibiotics for *Clostridium difficile* infection^56^ (particularly to reduce recurrence), its ability to affect tumors located outside the gut has not previously been demonstrated. Using a multi-omics approach, we show that *S. boulardii* alters circulating metabolites and cytokines and induces coordinated changes in tumor gene expression programs associated with disease progression. Targeted LC–MS/MS of spent *S. boulardii* cultures further supported these observations by confirming production of 5-HIAA, but not IPA, under experimental conditions, consistent with the idea that changes in circulating indole metabolites arise from both direct and microbiome-mediated effects. In antibiotic-perturbed human gut microbiota models, *S. boulardii* CNCM I-745 also did not produce IPA but instead facilitated the recovery of bacterial taxa responsible for IPA synthesis, leading to the reappearance of this metabolite as the community rebounded.^57^ Together, these findings indicate that elevated IPA is unlikely to originate from *S. boulardii* itself and more plausibly reflects an indirect, microbiome-mediated effect. Given our observations that different *S. boulardii* strains and products consistently activate AhR *in vitro* (Figure 1A), and recent genomic and functional comparisons across five strains reveal highly similar properties,^58^ these features appear broadly shared across *S. boulardii*. However, because our *in vivo* experiments used only the DBVPG 6763 strain, further work is needed to determine whether the tumor-suppressive effects observed here extend to other strains.

Previous studies have shown that *S. boulardii* limits tumor development in inflammation-driven CRC models by reducing local inflammation and preserving epithelial barrier integrity. For example, *S. boulardii* administration in AOM/DSS-treated mice reduced tumor burden, inflammatory cytokines, and epithelial damage.^59^ In ulcerative colitis–associated carcinogenesis models, treatment with this probiotic likewise lowered tumor numbers and TNF-*α* and IL-6 levels.^60^ In APC^Min^ mice, *S. boulardii* suppressed tumor growth by inactivating EGFR signaling in epithelial cells.^61^ Together, these studies establish a protective role for *S. boulardii* within the intestinal tumor microenvironment; here, we extend this work by showing that the yeast can also restrain tumor growth at a distant site, consistent with a systemic mode of action. By integrating metabolomics, cytokine profiling, and tumor RNA-sequencing, we provide a comprehensive view of how a probiotic can shape host physiology to influence tumor behavior. This expands on recent studies showing that gut-derived microbial metabolites, including AhR ligands, can reach distal tissues and alter immune signaling.^62^ ^,^ ^63^

In addition to systemic effects, *S. boulardii* caused significant changes in the gut microbiota, despite not being a long-term colonizer in mice^64^ or humans.^65^ Taxonomic profiling revealed a lower Firmicutes/Bacteroidetes ratio, opposite to the elevated ratios commonly reported in CRC,^66-68^ indicating a shift toward a composition closer to healthy gut states. At the functional level, pathways related to biosynthesis of amino acids, carbohydrate metabolism and fermentation (e.g., glycolysis and pyruvate fermentation) decreased, while nucleotide biosynthesis pathways were enriched. Genome‑resolved analyzes of colonic metagenomes showed that *S. boulardii* supplementation supports the growth of taxa with lower metabolic independence. Together with prior evidence that *S. boulardii* donates amino acids in co‑culture,^25^ this pattern is consistent with extracellular metabolite provisioning that relaxes selection for biosynthetic self‑sufficiency and favors expansion of LMI species. These changes suggest a remodeling of microbial metabolism that could alter the pool of circulating metabolites, although our data do not establish how this metabolic-independence axis contributes to downstream metabolite production or anti-tumor immunity. Although we did not causally link specific taxa or pathways to tumor outcomes, our data suggest that transient microbiome reshaping can initiate systemic immunometabolic changes that influence tumor progression.

*S. boulardii* administration reduced systemic IL-17A, consistent with evidence that microbiota-derived ligands can shift T cell polarization away from pro-tumorigenic Th17 cells toward less inflammatory states.^69^ For instance, a recent study demonstrated that *L. reuteri* expanded by statin treatment produces ILA, which downregulates IL-17 signaling in CRC by directly inhibiting Th17 cell differentiation (by antagonizing RORγt).^6^ Similarly, probiotic treatments have been shown to reduce intratumoral Th17 cell frequencies and IL-17A levels. For example, a multi-strain probiotic regimen markedly decreased IL-17 production and Th17 accumulation in a hepatocellular carcinoma model, impeding tumor growth.^70^ Notably, the reduced intratumoral Th17 cell count was attributed to diminished Th17 migration from the gut periphery. Together, these observations of IL-17 suppression highlight an emerging paradigm where microbial factors exert anti-tumor effects by restraining the pathogenic IL-17/Th17 axis.

Shifts in plasma cytokines and metabolites converge on the tumor itself, where we observe coordinated changes in gene expression programs. Although relatively few individual genes were significantly differentially expressed, gene set enrichment analysis revealed consistent downregulation of transcriptional programs involved in proliferation, invasion, and immune evasion. Beyond these pathway-level trends, PREDA analysis showed that many of the subtle transcriptional changes were spatially clustered, identifying contiguous genomic regions with coordinated downregulation rather than isolated single-gene effects. Genes within these PREDA-defined regions formed densely connected functional modules in STRING, enriched for translation, metabolic processes, and ER and Golgi trafficking. These patterns indicate that the regional suppression detected by PREDA corresponds to biologically coherent networks rather than random clustering. Consistent with the increase in circulating AhR ligands, tumors from *S. boulardii*–treated mice also showed a modest increase in the mean expression of AhR transcriptional targets. Together, these transcriptomic features complement the systemic reduction in IL-17A and CTLA-4 and are consistent with indirect modulation of tumor-associated signaling following *S. boulardii* treatment. Several of the suppressed pathways, including inflammatory response, IL6–JAK–STAT3 signaling, and TNF-*α* signaling, are regulated by NF-κB, a transcription factor with a central role in inflammation-driven tumor progression. Previous work has shown that *S. boulardii* inhibits NF-κB activation by preventing nuclear translocation of p65,^71^ which attenuates downstream inflammatory gene expression. This mechanism is consistent with the pathway-level suppression observed in our data.^71^

Multi-omics analysis showed that *S. boulardii* treatment led to consistent changes across the microbiome, circulating metabolites, immune signaling, and tumor gene expression. These changes were not only distinct within each dataset but also correlated across blocks, indicating a coordinated biological response. This supports the idea that *S. boulardii* is associated with changes across multiple interconnected pathways, linking microbial shifts to systemic and tumor-level effects. Importantly, the multi-omics integration identifies coordinated cross-compartment responses but does not establish directionality or mechanistic hierarchy among these layers.

In clinical settings, *S. boulardii* has already been tested perioperatively in colorectal surgery; in a randomized trial (*n* = 33), ≥7 days of preoperative oral *S. boulardii* reduced colonic mucosal mRNA levels of IL-1β, IL-10, and IL-23A, demonstrating probiotic-driven modulation of the local cytokine milieu in humans undergoing colorectal resection.^42^ In our study, oral *S. boulardii* lowered circulating IL-17A and CTLA-4, and in the TCGA combined COAD/READ cohort, patients with lower tumor IL-17A and CTLA-4 expression showed improved overall survival. These observations suggest that *S. boulardii* may influence tumor-relevant immunometabolic pathways in ways that could be therapeutically useful.

Although this study provides a comprehensive view of how S. boulardii influences the microbiome, systemic metabolism, immune signaling, and tumor transcriptional programs, several limitations should be acknowledged.

First, the mechanistic relationships linking these compartments remain correlative. Randomization supports that S. boulardii caused the observed treatment-associated changes in the microbiome, plasma metabolites, cytokines, tumor transcriptional programs, and tumor growth. However, the present design does not resolve the sequence of events connecting these layers. It therefore remains unclear whether microbiome remodeling initiates downstream metabolic and immune changes, whether tumor-derived factors feed back to shape the microbiota, or whether several of these processes occur in parallel. Dissecting these relationships will require experimental designs that decouple probiotic exposure from community restructuring, including germ-free systems, fecal microbiota transplantation with washout paradigms, or targeted strain-level manipulation.

Second, although our data support AhR pathway engagement, they do not establish that AhR signaling is necessary or sufficient for the observed reduction in tumor growth. The evidence for AhR involvement includes the *in vitro* reporter activity of S. boulardii-conditioned medium, elevated circulating AhR agonists, and a modest increase in tumor expression of an AhR target-gene set. These findings are consistent with increased AhR pathway activity, but they do not prove direct AhR regulation within tumor cells, nor do they demonstrate that AhR activation is required for tumor suppression. Future studies should directly test this by using AhR-deficient host mice, AhR-deficient tumor cells, or pharmacological AhR inhibition. Conversely, sufficiency could be addressed by testing whether exogenous 5-HIAA, IPA, or defined metabolite combinations recapitulate the metabolic, immune, transcriptional, and tumor-growth phenotypes observed after S. boulardii treatment. AhR biology is also not fully conserved between mice and humans, with species-specific differences in ligand sensitivity and transcriptional responses.^72^ Dedicated validation will therefore be required to determine how these findings translate to human CRC biology.

Third, the use of a subcutaneous syngeneic MC38 transplant model was intended to test whether oral *S. boulardii* supplementation can influence a distal tumor site through systemic metabolic and immune changes. This design is useful for separating systemic effects from direct local interactions between the gut lumen, intestinal epithelium, and tumor. However, it does not recapitulate the anatomical context of colorectal cancer or local tumor–microbiome interactions within the intestine. Moreover, because the anti-tumor effect was tested in a single syngeneic tumor model, it remains unclear whether this response reflects an MC38-specific phenotype or a broader effect across colorectal cancer models. Validation in additional syngeneic models, as well as orthotopic, genetically engineered, or inflammation-driven colorectal cancer models, will therefore be important to determine how *S. boulardii*-associated systemic effects interact with local epithelial, microbial, and immune processes during intestinal tumorigenesis.

Fourth, several of the microbiome and immune findings should be interpreted as associations rather than functionally validated mediators. The enrichment of low metabolic independence taxa is a genome-resolved ecological signature of *S. boulardii*-associated microbiome remodeling, but our data do not show that these taxa are necessary or sufficient for tumor suppression. Similarly, changes in circulating IL-17A, CTLA-4, and CCL11 indicate systemic immune modulation, but plasma cytokine abundance is not equivalent to tumor-local immune-cell composition. Bulk RNA-seq deconvolution did not identify robust differences in major tumor immune-cell populations, and subtle, spatially restricted, or functionally distinct immune changes arenot detectable with this approach. Future work should combine targeted microbial perturbation with higher-resolution immune profiling, including flow cytometry, single-cell RNA-seq, spatial transcriptomics, or histological analysis.

Together, these limitations mean that the present study should be interpreted as identifying coordinated microbiome, metabolic, immune, and tumor-transcriptional changes accompanying *S. boulardii*-associated tumor suppression, rather than as definitive proof of a single causal mechanism. The study establishes a framework and a set of testable hypotheses for future mechanistic work, including direct assessment of AhR requirement, metabolite sufficiency, microbiome transferability, validation across additional syngeneic tumor models, and validation in anatomical colorectal cancer models.

## Materials and methods

### Probiotic growth and spent media preparation for AhR activity screening

All probiotic products (Supplementary Table 1) were tested under both aerobic and anaerobic conditions. For anaerobic growth, MRS broth (Lactobacillus Broth according to DE MAN, ROGOSA and SHARPE, Sigma-Aldrich catalog number 1.10661.0500) was pre-reduced in a Whitley H35 Hypoxystation (Don Whitley Scientific) for 48 hours. Then, 2 mL of fresh MRS (pre-reduced for anaerobic conditions or kept at ambient conditions for aerobic growth) was inoculated with each probiotic product and incubated at 37 °C for 48 hours under static conditions. After incubation, optical density was measured at 600 nm, and cultures were centrifuged at 8,000 × g for 10 min at 4 °C. The spent media was collected and filter-sterilized through a 0.4 µm syringe filter. Spent medium from uninoculated MRS broth, treated identically, served as the negative control. The resulting spent media were used in the AhR activity assay and analysis of indole metabolites.

### Cell culture and maintenance

The murine colorectal MC38 cell line was kindly provided by Professor Janine Erler, Copenhagen University. Luciferase-labeled HT29-Lucia™ cells with AhR driven by the Cyp1a1 gene promoter (referred to as AhR reporter cells), were acquired from InvivoGen (catalog reference ht2l-ahr). MC38 cells were grown in RPMI 1640 Medium (Thermo Fisher Scientific, catalog number 11875093) and HT29-Lucia cells in McCoy’s 5 A (Modified) Medium (Thermo Fisher Scientific, catalog number 16600082). Media was supplemented with 10% fetal bovine serum (Thermo Fisher Scientific, catalog number A5670701) and 1% penicillin-streptomycin (Thermo Fisher Scientific, catalog number 15140148) in a humidified incubator at 37 °C with 5% CO_2_. Cells were maintained by changing the media twice per week and passaged upon reaching 90% confluency to ensure optimal growth conditions and cellular health. All cell lines were tested for the presence of mycoplasma to ensure their integrity and suitability for experimental use. For animal experiments, three generation old MC38 cells were prepared for subcutaneous injection by washing in-cold PBS three times and resuspended in serum-free medium.

### AhR activity assay

HT29-Lucia™ AhR Cells were handled according to the manufacturer’s instructions. Briefly, cells were thawed and passaged for two generations without the selection antibiotic zeocin (InvivoGen CAS number 11006-33-0). Subsequent passages were maintained in 100 µg/mL zeocin and 1% penicillin-streptomycin. Cells were harvested before reaching 90% confluency, and approximately 5000 cells per well were seeded in 96-well plates (Greiner bio-one catalog number 655160) in 180 µL volume. 20 µL of sample or positive control (6-formylindolo[3,2-b]carbazole (FICZ; Sigma-Aldrich, catalog number 172922-91-7)) was then added to a final volume of 200 µL and incubated for 48 hours. Following incubation, 20 µL of stimulated cell supernatant was transferred to a black 96-well plate with an optical bottom (Thermo Fisher Scientific catalog number 165305). To each well, 50 µL QUANTI-Luc™: Luciferase Detection Reagent (InvivoGen catalog number rep-qlc4r2) was added, and luminescence was immediately read on a Synergy H1 plate reader (BioTek) using 2 mm read-height, 200 ms integration time and 100 gain.

### Preparation of S. boulardii for oral gavage

Cryopreserved *S. boulardii* DBVPG 6763 were streaked onto yeast extract peptone dextrose (YPD) agar plates and incubated appropriately to obtain isolated colonies. Pre-cultures were initiated by inoculating three isolated colonies into 5 mL YPD medium, followed by incubation overnight at 37 °C. Subsequently, overnight cultures were transferred into 20 mL of fresh YPD medium in a shake flask and incubated for an additional 4 hours. After incubation, 1 mL of the resulting culture was evenly spread onto YPD agar plates and incubated for 48 hours. Cells were harvested by adding 1 mL of 1x PBS supplemented with 20% glycerol directly to the plate surface and scraping off the cell biomass. The collected cells were diluted to achieve an OD_600_ of 300, corresponding approximately to a concentration of 3 × 10^8^ CFU of *S. boulardii*.

### Animal experiments

Animal experiments were conducted in accordance with the Danish Animal Experiments Act on protection of animals used for scientific purpose (LBK 1107 from 02/07/2022) and Directive 2010/63/EU of the European Parliament. Moreover, the study protocols were approved by the Animal Experimentation Committee under the Ministry of Food, Fishing, and Agriculture (license number 2021-15-0201-00925). 39 female C57BL/6 (Taconic) seven-week-old underwent seven days of acclimatization with *ad libitum* access to tap water and chow diet (A30, Safe diets). 3 × 10^5^ MC38 (C57BL/6 mice) tumor cells in 100 µl serum-free media were subcutaneously engrafted on the right hind flank using a 26-gauge needle. Tumors were grown to an average volume of 150 mm^3^ before the mice were randomized into two groups (n ≥ 19). Mice received a single daily dose of 100 µL of 3 × 10^8^ CFU of *S. boulardii* DBVPG 6763 by intragastric gavage, or 100 µL of an empty vehicle dose consisting of 1 × PBS supplemented with 20% glycerol. Tumors were assessed three times per week using caliper and the volume was estimated using the formula 0.5 x (longest dimension x shortest dimension.^2^) After nine days of treatment, all mice were humanely euthanized using cervical dislocation. Whole blood was collected upon euthanizing and immediately processed using BD Biosciences Microtainer™ Tubes with Microgard™ Closure (Thermo Fisher Scientific product code 12957646) by centrifugation at 9000 x g for 2 minutes. Plasma was collected and immediately stored at -70 °C for further use. The tumor and spleen weights were recorded, and 50-100 mg of tumor was snap frozen and stored at -70 °C from all mice for bulk RNA-sequencing. The mice were co-housed in groups of 4-6 in individually ventilated cages (IVCs), 22 °C ± 2 °C, light cycle was 6 am to 6 pm, and had *ad libitum* access to water and chow diet (A30, Safe diets) during the study. The animal studies were carried out as single-blinded trials, with tumor measurements conducted by an animal caretaker who was blinded to treatment assignment.

### Plasma metabolomics

Sample analysis was carried out by MS-Omics. The analysis was carried out using a Thermo Scientific Vanquish LC coupled to an Orbitrap Exploris 240 MS, Thermo Fisher Scientific. An electrospray ionization interface was used as ionization source. Analysis was performed in positive and negative ionization mode under polarity switching. The UHPLC was performed using a slightly modified version of the protocol (UPLC/MS Monitoring of Water-Soluble Vitamin Bs in Cell Culture Media in Minutes, Water Application note 2011, 720004042en). Peak areas were extracted using Compound Discoverer 3.3 (Thermo Scientific). Identification of compounds was performed at four levels; Level 1: identification by retention times (compared against in-house authentic standards), accurate mass (with an accepted deviation of 3 ppm), and MS/MS spectra, Level 2a: identification by retention times (compared against in-house authentic standards), accurate mass (with an accepted deviation of 3 ppm). Level 2b: identification by accurate mass (with an accepted deviation of 3 ppm), and MS/MS spectra, Level 3: identification by accurate mass alone (with an accepted deviation of 3 ppm). Only compounds identified at level 1, 2a and 2b were considered for downstream analysis. Dimensionality reduction of the plasma metabolomics dataset was performed using the umap R package (v. 0.2.10.0). Numeric features were extracted from the dataset and reduced to two dimensions using UMAP with the following parameters: *n_neighbors* = 10, *min_dist* = 0.3, and *n_components* = 2. K-means clustering (k = 2) was applied to the UMAP output to define group centroids. Convex hulls were calculated using the chull algorithm to delineate cluster boundaries.

### Ultra-high performance liquid chromatography–tandem mass spectrometry analysis of indole metabolites

Spent medium from *S. boulardii* cultures was prepared by mixing 200 µL of clarified supernatant with 600 µL of ice-cold methanol, followed by incubation at –20 °C and centrifugation at 17,000 × g to remove precipitated material; 200 µL of the resulting supernatant was transferred to LC-MS vials for analysis. Commercial standards for indole derivatives were obtained from Sigma Aldrich (Merck Life Science A/S, Søborg, Denmark), including 5-hydroxyindole-3-acetic acid (5-HIAA), indole-3-acetic acid (IAA), indole-3-carboxaldehyde, indole-3-lactic acid (ILA), indole-3-propionic acid (IPA), 3-indoxylsulfate (indoxyl sulfate potassium salt), tryptophol (3-(2-hydroxyethyl)indole), indole-3-acetamide, indole-3-acrylic acid, and L-tryptophan. LC-MS grade acetonitrile (ACN) and water (H₂O) were purchased from Honeywell Research Chemicals (New Jersey, USA), and ≥99% formic acid (HCOOH) and absolute ethanol (EtOH) were obtained from VWR (Avantor, Radnor, PA, USA). Stock solutions of indole-3-carboxaldehyde, indole-3-acetic acid, tryptophol (3-(2-hydroxyethyl)indole), indole-3-acetamide, indole-3-acrylic acid, indole-3-propionic acid, indole-3-lactic acid, and 5-hydroxyindole-3-acetic acid were prepared in EtOH; the stock solution of L-tryptophan was prepared in H₂O:EtOH (50:50, v/v); the stock solution of indoxyl sulfate potassium salt (3-indoxylsulfate) was prepared in H₂O.

All stock solutions were diluted in H₂O containing 0.1% HCOOH to generate working standard solutions at 1 mg mL^−1^, which were further diluted in H₂O to prepare a matrix-matched calibration curve covering 0.1, 0.25, 0.5, 1, 2.5, 5, 10, 25, 50, 100, 250, 500, and 1000 ng mL^−1^.

Samples were analyzed on an ACQUITY Premier UPLC system (Waters Corp., Milford, MA, USA) coupled to a Xevo TQ Absolute triple quadrupole mass spectrometer (Waters) operated in electrospray ionization mode in both positive and negative ion modes. Selected reaction monitoring (SRM) was used for quantification, with cone voltages and collision energies individually optimized for each ion transition. High-purity nitrogen and argon served as source and collision cell gases, respectively. Chromatographic separation was performed using an ACQUITY UPLC® BEH C18 column (2.1 × 100 mm, 1.7 µm; Waters) held at 40 °C, with a flow rate of 0.35 mL min^−1^ and a binary solvent system consisting of H₂O + 0.1% (v/v) HCOOH (eluent A) and ACN + 0.1% (v/v) HCOOH (eluent B). The gradient program was: 0–0.1 min, 2–10% B; 0.1–10 min, 10–30% B; 10–10.1 min, 30–100% B; 10.1–12 min, 100% B; 12–12.1 min, 100–2% B, followed by a 2 min re-equilibration at 2% B. Injection volume was 2 µL for all samples. Data acquisition was performed using MassLynx 4.2 and processed with TargetLynx, and chrmatographic peak areas were used for quantification of all detected indole derivatives.

### Shotgun metagenomics on colon content

Metagenomic shotgun sequencing was performed by Novogene Co., Ltd using their standard protocol. Briefly, genomic DNA was extracted from each sample using validated methods. The extracted DNA was randomly sheared into shorter fragments and then end-repaired and A-tailed. Illumina-compatible adapters were ligated to the fragments. Size selection and purification of the adapter-ligated fragments were carried out prior to quality assessment by Qubit fluorometry, quantitative PCR, and fragment analysis. The qualified libraries were pooled based on their effective concentrations and sequenced on an Illumina platform, generating paired-end reads of 150 bp. Raw sequencing data were processed using Novogene’s quality control pipeline, which involved trimming adapter sequences, discarding reads containing more than 10% ambiguous bases (N’s), and filtering out reads with over 50% low-quality bases (Q-score ≤ 5). The resulting high-quality clean reads were used for subsequent downstream analyzes. To further clean the reads, we removed host-derived sequences by aligning the clean reads against the standard mouse reference genome (GRCm38/mm10) using Bowtie2^73^ with default parameters. Reads mapping to the mouse genome were discarded to eliminate potential contamination. The remaining non-host reads were further processed with Trimmomatic^74^ to trim any residual adapter sequences and low-quality bases. Taxonomic classification of metagenomic reads was performed using Kaiju^75^ (v1.8.2). Kaiju was run in greedy mode against the RefSeq genomes database. A low-abundance filter of 0.1% was applied to remove taxa with minimal representation across samples. Taxonomic classification was performed for multiple levels, including phylum, class, order, family, genus, and species. Kaiju output files were parsed, and taxonomic data were combined into a single dataset for downstream analysis. Taxonomic levels were standardized, and the resulting data were deduplicated to ensure unique taxa assignments per sample. Raw counts for each taxon were aggregated and transformed into relative abundances by dividing the counts for each taxon by the total counts per sample (Total-sum scaling). Alpha diversity assessed using Simpson diversity index (1-D) was calculated using the vegan R package (v2.6). Beta diversity was assessed using Bray-Curtis dissimilarities, computed with the vegan package, and principal coordinates analysis (PCoA) was performed on the Bray-Curtis distance matrix. PERMANOVA (adonis2) was applied to test for significant differences in community composition between treatment groups (999 permutations). Differential abundance between study groups was quantified using linear discriminant analysis effect size (LEfSe) analysis,^76^ performed using the LEfSe R package.^77^ Relative abundances were tested for significant differences between treatment groups at multiple taxonomic levels. Statistical thresholds were set at *p* < 0.05 for Kruskal-Wallis and Wilcoxon rank-sum tests, and LDA scores > 2.0 were used to identify enriched taxa. False discovery rate (FDR) correction was applied to account for multiple comparisons, ensuring robust identification of biomarkers. As LEfSe examines taxa individually and may overlook synergistic, non-linear interactions, we further employed an extreme gradient boosting (XGBoost) model to capture these complex relationships. The XGBoost model was implemented using the R package xgboost (version 1.7.8.1) to classify samples based on their microbial relative abundance profiles. The analysis utilized microbial taxa as features and the sample class (control or treatment) as the outcome variable. To ensure robust evaluation and prevent overfitting, we employed 5-fold cross-validation, where the data were split into training and validation subsets across folds, ensuring all samples were used for both training and testing. Hyperparameters were fixed based on initial optimization, including 500 boosting rounds, a learning rate (eta) of 0.01, a maximum tree depth of 3, gamma regularization of 1, a subsampling ratio of 0.7, and column sampling set to 1. The model’s performance was assessed using the area under the receiver operating characteristic (ROC) curve (AUC) as the primary metric, calculated for cross-validated predictions. Feature importance was determined based on three metrics: Gain, representing the relative improvement in predictive accuracy from splits on a feature; Cover, representing the proportion of samples affected by splits on a feature; and Frequency, representing the number of times a feature was used in splitting. These metrics enabled interpretation of the biological relevance of microbial taxa contributing to classification.

### Functional profiling using HUMAnN3

Functional profiling of metagenomic reads was conducted using the HMP Unified Metabolic Analysis Network version 3.0 (HUMAnN3) to characterize the metabolic potential of microbial communities. Pre-processed, high-quality metagenomic reads were aligned against the ChocoPhlAn 3 database to perform taxonomic profiling, followed by alignment to the UniRef90 protein sequence database for functional gene family identification. HUMAnN3 reconstructed metabolic pathways using the MetaCyc database, enabling a detailed characterization of microbial functions within each sample.

HUMAnN3 produced stratified gene-family and pathway abundance tables. Gene-family abundances were normalized as reads per kilobase to account for gene length. Pathway abundances were computed from the contribution of detected gene families to annotated metabolic pathways.Unmapped and unintegrated gene families and pathways were excluded to focus the analysis on confidently assigned functions. The output data were then normalized to relative abundance to facilitate cross-sample comparisons. Additionally, HUMAnN3 outputs were converted to Enzyme Commission numbers and Kyoto Encyclopedia of Genes and Genomes (KEGG) pathways using its internal conversion functions. This allowed for broader functional interpretation and cross-database comparisons. Stratified output data, indicating the specific microbial taxa contributing to each pathway, were retained for downstream analysis. The contribution of individual taxa to specific pathways was visualized using HUMAnN3’s internal barplot function, enabling detailed interpretation of pathway stratification across microbial species. A pseudocount of 1e-9 was added to all relative abundance values. Functional dissimilarity between samples was assessed by calculating Bray-Curtis dissimilarities using the vegdist function from the vegan R package (version 2.6). Principal Coordinates Analysis was performed on the Bray-Curtis distance matrix using the cmdscale function in R to visualize clustering patterns based on functional composition. Differentially abundant pathways and gene families between treatment groups were identified using linear discriminant analysis effect size. The analysis employed Kruskal-Wallis and Wilcoxon rank-sum tests with a *p*-value threshold of 0.05, and features were considered significantly enriched if their discriminant analysis score exceeded 2.0.

### Metagenome assembled genomes and metabolic independence scoring

Raw metagenomic reads were quality-filtered and adapter-trimmed with fastp v1.0.1^78^ (default settings). Per-sample assemblies were generated using MEGAHIT v1.2.9^79^ producing one contig set per sample. Binning of assembled contigs was performed with MetaBAT2 v2.18 ^80^ using default parameters. MAG quality was assessed with CheckM v1.2.4^81^ (lineage_wf), and bins meeting ≥90% completeness and ≤5% contamination were retained for downstream analyzes. High-quality MAG FASTA files were processed in anvi’o v8^82^ using the contigs workflow to create contigs.db databases, followed by HMMs, KEGG KOfam annotations, and single-copy gene taxonomy. Genome-level taxonomy was assigned with anvi-estimate-scg-taxonomy, which leverages the GTDB-Tk database.^83^ KEGG module completeness per MAG was computed via anvi-estimate-metabolism, and metabolic independence was called using anvi-script-estimate-metabolic-independence^39^ with the default 33-module panel and default settings. All steps were executed with default parameters unless otherwise stated, and only the procedures listed above contributed to the final results.

### RNA-sequencing of tumor tissue

Total RNA was extracted from subcutaneous tumors of mice treated with PBS or *S. boulardii*. RNA integrity was assessed using an Agilent Bioanalyzer, and libraries were prepared using the Illumina TruSeq Stranded mRNA protocol. Paired-end sequencing (2 × 150 bp) was performed on an Illumina NovaSeq 6000 platform. Raw sequencing reads were processed using the nf-core/rnaseq pipeline (v3.11.2) executed with Nextflow (v23.10.0) on a high-performance computing cluster configured with Singularity containers to ensure reproducibility. The pipeline was run with the --aligner star_salmon option, enabling alignment with STAR and quantification with Salmon in selective alignment mode. Reference genome and annotation files for Mus musculus (GRCm39) were provided via the --fasta and --gtf parameters, respectively, and a decoy-aware transcriptome index was generated for Salmon. Adapter trimming was performed using Trim Galore, and quality control metrics were aggregated using MultiQC. Strandedness was inferred automatically by the pipeline using Salmon's built-in functionality. Gene-level counts were extracted from the salmon.merged.gene_counts.tsv output file. Genes with fewer than 10 counts across all samples were excluded from downstream analysis. The count matrix was imported into R (v4.3.1) and processed with the DESeq2 package (v1.40.2). Differential expression analysis was conducted using the Wald test, with the treatment group as the design variable and independent filtering disabled to retain all genes for downstream analysis. Adjusted *p*-values were calculated using the Benjamini-Hochberg method, and genes with padj < 0.1 were considered significantly differentially expressed. For gene set enrichment analysis (GSEA), genes were ranked by log₂ fold change without significance filtering and analyzed using the clusterProfiler package (v4.10.1), with 20,000 permutations and scoretype = “std” and eps = 0 to enhance precision and allow estimation of extremely low *p*-values. Mouse-native Hallmark gene sets were retrieved using msigdbr (v7.5.1) with db_species = “MM” and mapped to Ensembl IDs using org.Mm.eg.db. Pathways with adjusted *p*-values < 0.1 were defined as significantly enriched. Shared leading-edge genes were evaluated by constructing a binary gene-by-pathway matrix and plotting intersections using UpSetR. To assess whether groups of spatially adjacent genes exhibited coordinated expression changes, we applied the PREDA^84^ (v1.54.0) framework. Gene coordinates were obtained by constructing a TxDb object from the Ensembl GRCm39 GTF using txdbmaker and GenomicFeatures. DESeq2 Wald statistics were extracted for all genes and formatted for PREDA using *StatisticsForPREDAFromdataframe*. Genomic annotations were generated from the GTF-derived coordinates with *GenomicAnnotationsFromdataframe*, using per-gene median chromosomal positions as reference coordinates. Genes on chromosomes 1–19 and X were retained, and annotations and statistics were merged using *MergeStatisticAnnotations2DataForPREDA*. PREDA smoothing and permutation testing were performed with *PREDA_main* (nperms = 1000; lokern_scaledBandwidthFactor = 2), and significant genomic regions were identified with *PREDAResults2GenomicRegionsSingle* using an FDR threshold of 0.10 and upper or lower tails corresponding to up- or down-regulated regions. Resulting regions were converted to genomic intervals and visualized as chromosome-wise segment plots. Genes overlapping PREDA-defined downregulated genomic regions were identified by intersecting significant genomic intervals with GTF-derived gene coordinates using GenomicRanges. These genes were mapped to STRING identifiers using STRINGdb (v2.10.0; species = *Mus musculus*, version 12; score_threshold = 400). Protein–protein interaction networks were retrieved from STRING and constructed as igraph objects, followed by Louvain community detection to identify densely connected clusters. Degree and betweenness centrality were calculated for all nodes. For each cluster, functional enrichment was obtained using STRINGdb’s built-in enrichment tables, including GO, KEGG, Reactome, and UniProt keyword categories. Cluster-level annotations were assigned based on the most strongly enriched term (lowest FDR). Networks were visualized with ggraph using Fruchterman–Reingold layout, with node size proportional to degree and node fill color corresponding to functional cluster labels.

### Plasma cytokine profiling

Plasma biomarkers were determined using proximity extension assay technology (Olink Proteomics, Inc.) on an Olink Target 48 Mouse Cytokine panel. Biomarkers with more than 20% of measurements outside of the assay limit of detection were excluded from the analysis.

### The cancer genome atlas (TCGA) analysis

Gene expression and clinical metadata from colorectal adenocarcinoma (COAD) and rectal adenocarcinoma (READ), were downloaded from The Cancer Genome Atlas (TCGA) using the TCGAbiolinks R package (v2.28). RNA-seq expression data were obtained as STAR-aligned counts. Genes of interest, specifically IL17A, CTLA4, and CCL11, were identified using their Ensembl gene identifiers. Normalized expression data were extracted, and samples were stratified into high and low expression groups based on a median split of gene expression levels. Clinical metadata, including survival information, were merged with the expression data using patient barcodes. Overall survival was calculated as the number of days from the date of diagnosis to either the date of death or the date of last follow-up. Patients who were alive at the last follow-up were considered censored observations. Kaplan-Meier survival curves were generated for each gene to compare survival between high and low expression groups. The survminer R package (v0.4.9) was used to visualize the survival curves, and the log-rank test was applied to assess statistical significance. Due to limited sample sizes in certain treatment categories, treatment type was not included as a covariate in the survival models.

### Multi-Omics integration using sparse partial least squares discriminant analysis (sPLS-DA)

Multi-omics integration was conducted using the DIABLO framework implemented in the *mixOmics* R package (version 6.30.0) under R version 4.4.2. Integration was restricted to mice with complete sample availability across metabolomics, microbiome, Olink cytokine profiling, and tumor RNA-seq datasets, resulting in 16 mice per group. The retained subset remained balanced for baseline tumor volume (PBS: 162.17 ± 94.38 mm³; S. boulardii: 168.68 ± 70.08 mm³; Welch’s t-test, *p* = 0.826) and baseline body weight (PBS: 19.07 ± 1.26 g; S. boulardii: 19.61 ± 0.86 g; Welch’s t-test, *p* = 0.169). Metabolomics, microbiome, cytokine, and RNA sequencing datasets were aligned by shared sample identifiers, and only mice present across all omics layers were retained for analysis. Pre-processing included the removal of zero- and near-zero variance features using the *caret* package (version 7.0-1). Missing values in the cytokine block were imputed using K-nearest neighbors (KNN) imputation from the *impute* package (version 1.80.0). All omics blocks were coerced into numeric matrices, and sample ordering was harmonized. A design matrix specifying equal correlation strength between blocks (off-diagonal entries set to 1, diagonal to 0) was used to define inter-block relationships. The treatment group was used as the outcome variable and encoded as a factor. Model tuning was performed using tune.block.splsda() with 5-fold cross-validation repeated 10 times, testing a grid of keepX values for each block over two components. The optimal number of features per block and component was selected based on classification performance. A final DIABLO model was trained using block.splsda() with the selected keepX values and the specified design. Model performance was evaluated using repeated 10-fold cross-validation via the perf() function, reporting classification error rates for both majority and weighted voting. ROC and AUC metrics were computed using the auroc() function to assess discriminative performance within individual blocks.

## Supplementary Material

Supplemental Material Supplementary Table 1

Supplemental Material Supplementary Table 2

Supplemental Material Supplementary Table 3

Supplementary_figure.docx Supplemental Material

## Data Availability

The RNA-sequencing and metagenomic datasets generated in this study have been deposited in the NCBI Sequence Read Archive (SRA) under the BioProject accession number PRJNA1313482.

## References

[1] Zitvogel L , Ma Y , Raoult D , Kroemer G , Gajewski TF . The microbiome in cancer immunotherapy: diagnostic tools and therapeutic strategies. Sci. 2018;359:1366–1370. doi: 10.1126/science.aar6918. 29567708

[2] Gopalakrishnan V , Spencer CN , Nezi L , Reuben A , Andrews MC , Karpinets TV , Prieto PA , Vicente D , Hoffman K , Wei SC , et al. Gut microbiome modulates response to anti-PD-1 immunotherapy in melanoma patients. Sci. 2018;359:97–103. doi: 10.1126/science.aan4236. PMC5827966 29097493

[3] Gutiérrez-Vázquez C , Quintana FJ . Regulation of the immune response by the aryl hydrocarbon receptor. Immunity. 2018;48:19–33. doi: 10.1016/j.immuni.2017.12.012. 29343438 PMC5777317

[4] Zelante T , Iannitti RG , Cunha C , De Luca A , Giovannini G , Pieraccini G , Zecchi R , D’Angelo C , Massi-Benedetti C , Fallarino F , et al. Tryptophan catabolites from microbiota engage aryl hydrocarbon receptor and balance mucosal reactivity via interleukin-22. Immunity. 2013;39:372–385. doi: 10.1016/j.immuni.2013.08.003. 23973224

[5] Jia D , Kuang Z , Wang L . The role of microbial indole metabolites in tumor. Gut Microbes. 2024;16:2409209. doi: 10.1080/19490976.2024.2409209. 39353090 PMC11445886

[6] Han JX , Tao Z , Wang J , Zhang L , Yu C , Kang Z , Xie Y , Li J , Lu S , Cui Y , et al. Microbiota-derived tryptophan catabolites mediate the chemopreventive effects of statins on colorectal cancer. Nature Microbiology 2023. 2023;8(5):919–933. doi: 10.1038/s41564-023-01363-5. 37069401

[7] Vaaben TH , Lützhøft DO , Koulouktsis A , Dawoodi IM , Stavnsbjerg C , Kvich L , Gögenur I , Vazquez-Uribe R , Sommer MOA . Modulating tumor immunity using advanced microbiome therapeutics producing an indole metabolite. EMBO Rep. 2025;26:1688–1708. doi: 10.1038/s44319-025-00386-9. 40055466 PMC11977207

[8] Redenti A , Im J , Li F , Rouanne M , Sheng Z , Sun W , Gurbatri CR , Huang S , Komaranchath M , Jang Y , et al. Probiotic neoantigen delivery vectors for precision cancer immunotherapy. Nature 2024. 2024;635(8038):453–461. doi: 10.1038/s41586-024-08033-4. PMC11560847 39415001

[9] Tumas S , Meldgaard TS , Vaaben TH , Suarez Hernandez S , Rasmussen AT , Vazquez-Uribe R , Hadrup SR , Sommer MOA . Engineered E. Coli nissle 1917 for delivery of bioactive IL-2 for cancer immunotherapy. Sci Rep. 2023;13:1–11. doi: 10.1038/s41598-023-39365-2. 37532747 PMC10397246

[10] Śliżewska K , Markowiak-Kopeć P , Śliżewska W . The role of probiotics in cancer prevention. Cancers (Basel). 2020;13:20. 33374549 10.3390/cancers13010020 PMC7793079

[11] Baruch EN , Youngster I , Ben-Betzalel G , Ortenberg R , Lahat A , Katz L , Adler K , Dick-Necula D , Raskin S , Bloch N , et al. Fecal microbiota transplant promotes response in immunotherapy-refractory melanoma patients. Sci. 2021;371:602–609. doi: 10.1126/science.abb5920. 33303685

[12] Davar D , Dzutsev AK , McCulloch JA , Rodrigues RR , Chauvin J , Morrison RM , Deblasio RN , Menna C , Ding Q , Pagliano O , et al. Fecal microbiota transplant overcomes resistance to anti-PD-1 therapy in melanoma patients. Sci. 2021;371:595–602. doi: 10.1126/science.abf3363. PMC8097968 33542131

[13] Mego M , Danis R , Chovanec J , Jurisova S , Bystricky B , Porsok S , Konkolovsky P , Vaclav V , Wagnerova M , Streško M , et al. Randomized double-blind, placebo-controlled multicenter phase III study of prevention of irinotecan-induced diarrhea by a probiotic mixture containing bifidobacterium BB-12® lactobacillus rhamnosus LGG® in colorectal cancer patients. Front Oncol. 2023;13:1168654. doi: 10.3389/fonc.2023.1168654. 37601667 PMC10438450

[14] Dizman N , Meza L , Bergerot P , Alcantara M , Dorff T , Lyou Y , Frankel P , Cui Y , Mira V , Llamas M , et al. Nivolumab plus ipilimumab with or without live bacterial supplementation in metastatic renal cell carcinoma: a randomized phase 1 trial. Nature Medicine 2022. 2022;28(4):704–712. doi: 10.1038/s41591-022-01694-6. PMC9018425 35228755

[15] Goldenberg JZ , Yap C , Lytvyn L , Lo CK , Beardsley J , Mertz D , Johnston BC . Probiotics for the prevention of clostridium difficile-associated diarrhea in adults and children. Cochrane Database Syst Rev. 2017;12. doi: 10.1002/14651858.CD006095.pub4. PMC6486212 29257353

[16] Rafter J , Bennett M , Caderni G , Clune Y , Hughes R , Karlsson PC , Klinder A , O'Riordan M , O'Sullivan GC , Pool-Zobel B , et al. Dietary synbiotics reduce cancer risk factors in polypectomized and colon cancer patients. Am J Clin Nutr. 2007;85:488–496. doi: 10.1093/ajcn/85.2.488. 17284748

[17] Tomita Y , Ikeda T , Sakata S , Saruwatari K , Sato R , Iyama S , Jodai T , Akaike K , Ishizuka S , Saeki S , et al. Association of probiotic clostridium butyricum therapy with survival and response to immune checkpoint blockade in patients with lung cancer. Cancer Immunol Res. 2020;8:1236–1242. doi: 10.1158/2326-6066.CIR-20-0051. 32665261

[18] Xia C , Jiang C , Li W , Wei J , Hong H , Feng L , Xin H , Chen T . A phase II randomized clinical trial and mechanistic studies using improved probiotics to prevent oral mucositis induced by concurrent radiotherapy and chemotherapy in nasopharyngeal carcinoma. Front Immunol. 2021;12:618150. doi: 10.3389/fimmu.2021.618150. 33841399 PMC8024544

[19] Mizuta M , Endo I , Yamamoto S , Inokawa H , Kubo M , Udaka T , Sogabe O , Maeda H , Shirakawa K , Okazaki E , et al. Perioperative supplementation with bifidobacteria improves postoperative nutritional recovery, inflammatory response, and fecal microbiota in patients undergoing colorectal surgery: a prospective, randomized clinical trial. Biosci Microbiota Food Health. 2016;35:77–87. doi: 10.12938/bmfh.2015-017. 27200261 PMC4858881

[20] Ahrén IL , Bjurberg M , Steineck G , Bergmark K , Jeppsson B . Decreasing the adverse effects in pelvic radiation therapy: a randomized controlled trial evaluating the use of probiotics. Adv Radiat Oncol. 2022;8:101089. doi: 10.1016/j.adro.2022.101089. 36483069 PMC9723296

[21] Juan Z , Chen J , Ding B , Yongping L , Cai H , Wang L , Le Y , Shi J , Wu Y , Ma D , et al. Probiotics prevent pegylated liposomal doxorubicin-associated hand-foot syndrome and oral mucositis of breast cancer patients following surgery and chemotherapy: a randomized placebo-controlled trial. Int J Surg. 2025;111:2018–2030. doi: 10.1097/JS9.0000000000002147. 39715143

[22] Wang X , Zhao D , Bi D , Li L , Tian H , Yin F , Zuo T , Ianiro G , Chen Q , Qin H . Fecal microbiota transplantation: transitioning from chaos and controversial realm to scientific precision era. Sci Bull (Beijing). 2025;70:970–985. doi: 10.1016/j.scib.2025.01.029. 39855927

[23] Spacova I , Binda S , ter Haar JA , Henoud S , Legrain-Raspaud S , Dekker J , Espadaler-Mazo J , Langella P , Martín R , Pane M , et al. Comparing technology and regulatory landscape of probiotics as food, dietary supplements and live biotherapeutics. Front Microbiol. 2023;14:1272754. doi: 10.3389/fmicb.2023.1272754. 38188575 PMC10770255

[24] Ciernikova S , Mego M , Semanova M , Wachsmannova L , Adamcikova Z , Stevurkova V , Drgona L , Zajac V . Probiotic survey in cancer patients treated in the outpatient department in a comprehensive cancer center. Integr Cancer Ther. 2017;16:188–195. doi: 10.1177/1534735416643828. 27151581 PMC5739119

[25] Hedin KA , Mirhakkak MH , Vaaben TH , Sands C , Pedersen M , Baker A , Vazquez-Uribe R , Schäuble S , Panagiotou G , Wellejus A , et al. Saccharomyces boulardii enhances anti-inflammatory effectors and AhR activation via metabolic interactions in probiotic communities. ISME J. 2024;18:212. doi: 10.1093/ismejo/wrae212. PMC11631509 39488793

[26] Vyhlídalová B , Krasulová K , Pečinková P , Marcalíková A , Vrzal R , Zemánková L , Vančo J , Trávníček Z , Vondráček J , Karasová M , et al. Gut microbial catabolites of tryptophan are ligands and agonists of the aryl hydrocarbon receptor: a detailed characterization. International Journal of Molecular Sciences 2020. 2020;21:2614 2614. doi: 10.3390/ijms21072614. PMC7177849 32283770

[27] Zeng L , Si Z , Zhao X , Feng P , Huang J , Long X , Yi Y . Metabolome analysis of the response and tolerance mechanisms of saccharomyces cerevisiae to formic acid stress. Int J Biochem Cell Biol. 2022;148:106236. doi: 10.1016/j.biocel.2022.106236. 35688405

[28] Fang CY , Chen J , Hsu B , Hussain B , Rathod J , Lee K . Colorectal cancer stage-specific fecal bacterial community fingerprinting of the Taiwanese population and underpinning of potential taxonomic biomarkers. Microorganisms. 2021;9:1548. doi: 10.3390/microorganisms9081548. 34442626 PMC8401100

[29] Heshiki Y , Vazquez-Uribe R , Li J , Ni Y , Quainoo S , Imamovic L , Sørensen M , Chow BKC , Weiss GJ , Xu A , et al. Predictable modulation of cancer treatment outcomes by the gut microbiota. Microbiome. 2020;8:1–14. doi: 10.1186/s40168-020-00811-2. 32138779 PMC7059390

[30] Segata N , Izard J , Waldron L , Gevers D , Miropolsky L , Garrett WS , Huttenhower C . Metagenomic biomarker discovery and explanation. Genome Biol. 2011;12:R60. doi: 10.1186/gb-2011-12-6-r60. 21702898 PMC3218848

[31] Huh JW , Kim MJ , Lee HG , Ryoo S , Ku J , Jeong S , Park KJ . Enterotypical prevotella and three novel bacterial biomarkers in preoperative stool predict the clinical outcome of colorectal cancer. Microbiome. 2022;10:203. doi: 10.1186/s40168-022-01388-8. 36443754 PMC9703702

[32] Bang S , Shin Y , Ma X , Park S , Graham DB , Xavier RJ , Clardy J . A cardiolipin from muribaculum intestinale induces antigen-specific cytokine responses. J Am Chem Soc. 2023;145:23422–23426. doi: 10.1021/jacs.3c09734. 37871232 PMC10623554

[33] Chen C , Wang J , Cheng M , Xie H , Li W , Zhang C . Muribaculum intestinale-derived 3-hydroxybutyric acid from heterophyllin B attenuated pulmonary fibrosis through IDO1-mediated ferroptosis. Pharmacol Res. 2025;212:107587. doi: 10.1016/j.phrs.2025.107587. 39778639

[34] Cong J , Liu P , Han Z , Ying W , Li C , Yang Y , Wang S , Cao F , Shen J , Zeng Y , et al. Bile acids modified by the intestinal microbiota promote colorectal cancer growth by suppressing CD8+ T cell effector functions. Immunity. 2024;57:876–889.e11. doi: 10.1016/j.immuni.2024.02.014. 38479384

[35] Wortmann E , Osswald A , Wylensek D , Kuhls S , Coleman O , Ducarmon Q , Liang W , Treichel N , Schumacher F , Volet C , et al. Secondary bile acid production by gut bacteria promotes Western diet-associated colorectal cancer. bioRxiv 2023. 2024;03(17):533140. doi: 10.1101/2023.03.17.533140. PMC13479223 41412727

[36] Byrd DA , Damerell V , Gomez Morales MF , Hogue SR , Lin T , Ose J , Himbert C , Ilozumba MN , Kahlert C , Shibata D , et al. The gut microbiome is associated with disease-free survival in stage I–III colorectal cancer patients. Int J Cancer. 2025;157:64–73. doi: 10.1002/ijc.35342. 39887373 PMC12262587

[37] Wu XQ , Ying F , Chung KPS , Leung CON , So KKH , Lei MML , Chau WK , Tong M , Yu J , Wei D , et al. Intestinal akkermansia muciniphila complements the efficacy of PD1 therapy in MAFLD-related hepatocellular carcinoma. Cell Rep Med. 2025;6:101900. doi: 10.1016/j.xcrm.2024.101900. 39798567 PMC11866522

[38] Li T , Lu X , Wu Y , Wu T , Xiao J . Metabolic influence of S. Boulardii and S. Cerevisiae in cross-kingdom models of S. Mutans and C. Albicans. Journal of Fungi. 2025;11:325. doi: 10.3390/jof11040325. 40278145 PMC12028775

[39] Veseli I , Chen YT , Schechter MS , Vanni C , Fogarty EC , Watson AR , Jabri B , Blekhman R , Willis AD , Yu MK , et al. Microbes with higher metabolic independence are enriched in human gut microbiomes under stress. eLife. 2025;12. doi: 10.7554/eLife.89862.3. PMC12084026 40377187

[40] Watson AR , Füssel J , Veseli I , DeLongchamp JZ , Silva M , Trigodet F , Lolans K , Shaiber A , Fogarty E , Runde JM , et al. Metabolic independence drives gut microbial colonization and resilience in health and disease. Genome Biology 2023. 2023;24(1):78. doi: 10.1186/s13059-023-02924-x. PMC10108530 37069665

[41] Zünd JN , Mujezinovic D , Reichlin M , Plüss S , Caflisch M , Robinson S , Lacroix C , Pugin B . Novel cross-feeding human gut microbes metabolizing tryptophan to indole-3-propionate. Gut Microbes. 2025;17. doi: 10.1080/19490976.2025.2501195. PMC12064059 40336187

[42] Consoli MLD , da Silva RS , Nicoli JR , Bruña‐Romero O , de Vasconcelos Generoso S , Correia MITD . Randomized clinical trial: impact of oral administration of saccharomyces boulardii on gene expression of intestinal cytokines in patients undergoing colon resection. Journal of Parenteral and Enteral Nutrition. 2016;40:1114–1121. doi: 10.1177/0148607115584387. 25917895

[43] Abbas Z , Yakoob J , Jafri W , Ahmad Z , Azam Z , Usman MW , Shamim S , Islam M . Cytokine and clinical response to saccharomyces boulardii therapy in diarrhea-dominant irritable bowel syndrome: a randomized trial. Eur J Gastroenterol Hepatol. 2014;26:630–639. doi: 10.1097/MEG.0000000000000094. 24722560

[44] Razi S , Baradaran Noveiry B , Keshavarz-Fathi M , Rezaei N . IL-17 and colorectal cancer: from carcinogenesis to treatment. Cytokine. 2019;116:7–12. doi: 10.1016/j.cyto.2018.12.021. 30684916

[45] Wang K , Kim MK , Di Caro G , Wong J , Shalapour S , Wan J , Zhang W , Zhong Z , Sanchez-Lopez E , Wu L , et al. Interleukin-17 receptor a signaling in transformed enterocytes promotes early colorectal tumorigenesis. Immunity. 2014;41:1052–1063. doi: 10.1016/j.immuni.2014.11.009. 25526314 PMC4272447

[46] Omura Y , Toiyama Y , Okugawa Y , Yin C , Shigemori T , Kusunoki K , Ide S , Shimura T , Fujikawa H , Yasuda H , et al. Prognostic impacts of tumoral expression and serum levels of PD-L1 and CTLA-4 in colorectal cancer patients. Cancer Immunol Immunother. 2020;69:2533–2546. doi: 10.1007/s00262-020-02645-1. 32577816 PMC11027465

[47] Zajkowska M , Kulczyńska-Przybik A , Dulewicz M , Safiejko K , Juchimiuk M , Konopko M , Kozłowski L , Mroczko B . Eotaxins and their receptor as biomarkers of colorectal cancer. J Clin Med. 2021;10:2675. doi: 10.3390/jcm10122675. 34204490 PMC8235018

[48] Weinstein JN , Collisson EA , Mills GB , Shaw KRM , Ozenberger BA , Ellrott K , Shmulevich I , Sander C , Stuart JM . The cancer genome Atlas pan-cancer analysis project. Nat Genet. 2013;45:1113–1120. doi: 10.1038/ng.2764. 24071849 PMC3919969

[49] Lonsdale J , Thomas J , Salvatore M , Phillips R , Lo E , Shad S , Hasz R , Walters G , Garcia F , Young N , et al. The genotype-tissue expression (GTEx) project. Nat Genet. 2013;45:580–585. doi: 10.1038/ng.2653. 23715323 PMC4010069

[50] Lachmann A , Xu H , Krishnan J , Berger SI , Mazloom AR , Ma'ayan A . ChEA: transcription factor regulation inferred from integrating genome-wide ChIP-X experiments. Bioinformatics. 2010;26:2438–2444. doi: 10.1093/bioinformatics/btq466. 20709693 PMC2944209

[51] Petitprez F , Levy S , Sun C , Meylan M , Linhard C , Becht E , Elarouci N , Tavel D , Roumenina LT , Ayadi M , et al. The murine microenvironment cell population counter method to estimate abundance of tissue-infiltrating immune and stromal cell populations in murine samples using gene expression. Genome Medicine 2020. 2020;12(1):86. doi: 10.1186/s13073-020-00783-w. PMC7541325 33023656

[52] Dupraz L , Magniez A , Rolhion N , Richard ML , Da Costa G , Touch S , Mayeur C , Planchais J , Agus A , Danne C , et al. Gut microbiota-derived short-chain fatty acids regulate IL-17 production by mouse and human intestinal γδ T cells. Cell Rep. 2021;36:109332. doi: 10.1016/j.celrep.2021.109332. 34233192

[53] Wang Y , Yin Y , Chen X , Zhao Y , Wu Y , Li Y , Xiang C . Induction of intestinal Th17 cells by flagellins from segmented filamentous bacteria. Front Immunol. 2019;10:466492. doi: 10.3389/fimmu.2019.02750. PMC6883716 31824516

[54] Singh A , Shannon CP , Gautier B , Rohart F , Vacher M , Tebbutt SJ , Lê Cao K , Birol I . DIABLO: an integrative approach for identifying key molecular drivers from multi-omics assays. Bioinformatics. 2019;35:3055–3062. doi: 10.1093/bioinformatics/bty1054. 30657866 PMC6735831

[55] Szajewska H , Kołodziej M . Systematic review with meta-analysis: saccharomyces boulardii in the prevention of antibiotic-associated diarrhoea. Aliment Pharmacol Ther. 2015;42:793–801. doi: 10.1111/apt.13344. 26216624

[56] Wombwell E . Saccharomyces boulardii prophylaxis for targeted antibiotics and infectious indications to reduce healthcare facility-onset clostridioides difficile infection. Microbes Infect. 2023;25:105041. doi: 10.1016/j.micinf.2022.105041. 36058513

[57] Huang Z , Brot L , Fatouh R , Bredon M , Creusot L , Lefèvre A , Lamazière A , Lefevre JH , Emond P , Planchais J , et al. Saccharomyces boulardii CNCM I-745 mitigates antibiotic-induced gut microbiome functional alterations independently of the host. Gut Microbes. 2025;17:2575924. doi: 10.1080/19490976.2025.2575924. 41200858 PMC12599567

[58] Duffey HE , Hedin KA , Gelli HP , Vaaben TH , Sommer MOA . Genomic and phenotypic comparison of saccharomyces cerevisiae and saccharomyces boulardii. bioRxiv. 2025. doi: 10.1101/2025.09.08.674931. PMC13141078 42095083

[59] Culpepper T , Senthil K , Vlcek J , Hazelton A , Heavey MK , Sellers RS , Nguyen J , Arthur JC . Engineered probiotic saccharomyces boulardii reduces colitis-associated colorectal cancer burden in mice. Dig Dis Sci. 2025;70:2348–2367. doi: 10.1007/S10620-025-09008-9. 40156662 PMC12301100

[60] Wang C , Li W , Ma Y , Zhao X , Zhang X , Yang H , Qian J . Saccharomyces boulardii alleviates ulcerative colitis carcinogenesis in mice by reducing TNF-α and IL-6 levels and functions and by rebalancing intestinal microbiota. BMC Microbiol. 2019;19:1–12. doi: 10.1186/s12866-019-1610-8. 31694526 PMC6836350

[61] Chen X , Fruehauf J , Goldsmith JD , Xu H , Katchar KK , Koon H , Zhao D , Kokkotou EG , Pothoulakis C , Kelly CP . Saccharomyces boulardii inhibits EGF receptor signaling and intestinal tumor growth in apcmin mice. Gastroenterology. 2009;137:914–923. doi: 10.1053/j.gastro.2009.05.050. 19482027 PMC2777664

[62] Hezaveh K , Shinde RS , Klötgen A , Halaby MJ , Lamorte S , Ciudad MT , Quevedo R , Neufeld L , Liu ZQ , Jin R , et al. Tryptophan-derived microbial metabolites activate the aryl hydrocarbon receptor in tumor-associated macrophages to suppress anti-tumor immunity. Immunity. 2022;55:324–340.e8. doi: 10.1016/j.immuni.2022.01.006. 35139353 PMC8888129

[63] Tintelnot J , Xu Y , Lesker TR , Schönlein M , Konczalla L , Giannou AD , Pelczar P , Kylies D , Puelles VG , Bielecka AA , et al. Microbiota-derived 3-IAA influences chemotherapy efficacy in pancreatic cancer. Nature 2023. 2023;615(7950):168–174. doi: 10.1038/s41586-023-05728-y. PMC9977685 36813961

[64] Hedin KA , Rees VE , Zhang H , Kruse V , Vazquez-Uribe R , Sommer MOA . Effects of broad-spectrum antibiotics on the colonisation of probiotic yeast saccharomyces boulardii in the murine gastrointestinal tract. Scientific Reports 2022. 2022;12(1):1–9. doi: 10.1038/s41598-022-12806-0. PMC9133042 35614092

[65] Klein SM , Elmer GW , McFarland LV , Surawicz CM , Levy RH . Recovery and elimination of the biotherapeutic agent, saccharomyces boulardii, in healthy human volunteers. SpringerSM Klein, GW Elmer, LV McFarland, CM Surawicz, RH LevyPharmaceutical research, 1993•Springer. 1993;10:1615–1619. 10.1023/a:1018924820333 8290474

[66] Liu J , Dong W , Zhao J , Wu J , Xia J , Xie S , Song X . Gut microbiota profiling variated during colorectal cancer development in mouse. BMC Genomics. 2022;23:1–13. doi: 10.1186/s12864-022-09008-3. 36550412 PMC9773433

[67] Kadhim FJ , Aziz ZS , Ibrahim KS . Gut microbiome profiles in colorectal cancer patients in Iraq. Microbiology Research 2025. 2025;16:22 22. doi: 10.3390/microbiolres16010022.

[68] Fang CY , Chen J , Hsu B , Hussain B , Rathod J , Lee K . Colorectal cancer stage-specific fecal bacterial community fingerprinting of the Taiwanese population and underpinning of potential taxonomic biomarkers. Microorganisms. 2021;9:1548. doi: 10.3390/microorganisms9081548. 34442626 PMC8401100

[69] Alexander M , Upadhyay V , Rock R , Ramirez L , Trepka K , Puchalska P , Orellana D , Ang QY , Whitty C , Turnbaugh JA , et al. A diet-dependent host metabolite shapes the gut microbiota to protect from autoimmunity. bioRxiv. 2024. doi: 10.1101/2023.11.02.565382. PMC11660937 39500329

[70] Li J , Sung CYJ , Lee N , Ni Y , Pihlajamäki Y , Panagiotou G , Nezami HL . Probiotics modulated gut microbiota suppresses hepatocellular carcinoma growth in mice. Proc Natl Acad Sci U S A. 2016;113:1306–E1315. 10.1073/pnas.1518189113 PMC4780612 26884164

[71] Sougioultzis S , Simeonidis S , Bhaskar KR , Chen X , Anton PM , Keates S , Pothoulakis C , Kelly CP . Saccharomyces boulardii produces a soluble anti-inflammatory factor that inhibits NF-κB-mediated IL-8 gene expression. Biochem Biophys Res Commun. 2006;343:69–76. doi: 10.1016/j.bbrc.2006.02.080. 16529714

[72] Xu X , Zhang X , Yuan Y , Zhao Y , Fares HM , Yang M , Wen Q , Taha R , Sun L . Species-specific differences in aryl hydrocarbon receptor responses: how and why? International Journal of Molecular Sciences 2021. 2021;22:13293 13293. doi: 10.3390/ijms222413293. PMC8708342 34948089

[73] Langmead B , Salzberg SL . Fast gapped-read alignment with bowtie 2. Nature Methods 2012. 2012;9(4):357–359. doi: 10.1038/nmeth.1923. PMC3322381 22388286

[74] Bolger AM , Lohse M , Usadel B . Trimmomatic: a flexible trimmer for illumina sequence data. Bioinformatics. 2014;30:2114–2120. doi: 10.1093/bioinformatics/btu170. 24695404 PMC4103590

[75] Menzel P , Ng KL , Krogh A . Fast and sensitive taxonomic classification for metagenomics with kaiju. Nature Communications 2016. 2016;7(1):1–9. doi: 10.1038/ncomms11257. PMC4833860 27071849

[76] Segata N , Izard J , Waldron L , Gevers D , Miropolsky L , Garrett WS , Huttenhower C . Metagenomic biomarker discovery and explanation. Genome Biol. 2011;12:R60. doi: 10.1186/gb-2011-12-6-r60. 21702898 PMC3218848

[77] Khleborodova A , Gamboa-Tuz SD , Ramos M , Segata N , Waldron L , Oh S . Lefser: implementation of metagenomic biomarker discovery tool. LEfSe, in R. Bioinformatics. 2024;40. 10.1093/bioinformatics/btae707 PMC11665633 39585730

[78] Chen S , Zhou Y , Chen Y , Gu J . Fastp: an ultra-fast all-in-one FASTQ preprocessor. Bioinformatics. 2018;34:i884–i890. doi: 10.1093/bioinformatics/bty560. 30423086 PMC6129281

[79] Li D , Liu C-M , Luo R , Sadakane K , Lam T-W . MEGAHIT: an ultra-fast single-node solution for large and complex metagenomics assembly via succinct *de bruijn* graph. Bioinformatics. 2015;31:1674–1676. doi: 10.1093/bioinformatics/btv033. 25609793

[80] Kang D , Li F , Kirton E , Thomas A , Egan R , An H , Wang Z . MetaBAT 2: an adaptive binning algorithm for robust and efficient genome reconstruction from metagenome assemblies. PeerJ 2019. 2019. e7359. doi: 10.7717/peerj.7359. PMC6662567 31388474

[81] Parks DH , Imelfort M , Skennerton CT , Hugenholtz P , Tyson GW . CheckM: assessing the quality of microbial genomes recovered from isolates, single cells, and metagenomes. Genome Res. 2015;25:1043–1055. doi: 10.1101/gr.186072.114. 25977477 PMC4484387

[82] Eren AM , Kiefl E , Shaiber A , Veseli I , Miller SE , Schechter MS , Fink I , Pan JN , Yousef M , Fogarty EC , et al. Community-led, integrated, reproducible multi-omics with anvi’o. Nat Microbiol. 2021;6:3–6. doi: 10.1038/s41564-020-00834-3. 33349678 PMC8116326

[83] Parks DH , Chuvochina M , Waite DW , Rinke C , Skarshewski A , Chaumeil P , Hugenholtz P . A standardized bacterial taxonomy based on genome phylogeny substantially revises the tree of life. Nat Biotechnol. 2018;36:996–1004. doi: 10.1038/nbt.4229. 30148503

[84] Ferrari F , Solari A , Battaglia C , Bicciato S . PREDA: an R-package to identify regional variations in genomic data. Bioinformatics. 2011;27:2446–2447. doi: 10.1093/bioinformatics/btr404. 21742634

