# Supplementary material for: Multi-omics analysis of saccharomyces boulardii supplementation reveals coordinated microbiome, metabolic, and immune signaling changes accompanying tumor suppression
Source: Gut Microbes. 2026 Jun 30;18(1):2690687. doi: 10.1080/19490976.2026.2690687 (PMC13336259; doi:10.1080/19490976.2026.2690687)
Supplement: Supplemental Material — Supplementary Table 1 [file KGMI_A_2690687_SM5728.docx]

Table 1 – Probiotic products screened for AhR activity

| Product Name | ID | Product Number | Product label |
| --- | --- | --- | --- |
| APOVIT DAGLIG MÆLKESYREBAKTERIER (30 STK.) | 1 | 222444 | Mælkesyrebakterier (Bifidobacterium animalis ssp. Lactis BIFOLAC 12, Bifidobacterium longum BB536, Lactococcus lactis LL-23, Lactobacillus acidophilus LA-14, Lactobacillus rhamnosus BIFOLAC GG), total: 5 mia CFU |
| DUOLAC BIFIDO+ DRÅBER | 2 | 225468 | Bifidobacterium infantis CBT BT1: 125 mio, Bifidobacterium breve CBT BR3: 125 mio, Bifidobacterium longum CBT BG7: 125 mio, Bifidobacterium bifidum CBT BF3: 125 mio |
| APOTEKETS D-DRÅBER MED MÆLKESYREBAKTERIER | 3 | 224874 | 6 dråber: 10 µg D-vitamin (200%); Lactobacillus rhamnosus GG: 670 mio |
| APOVIT REJSE MÆLKESYREBAKTERIER (10 STK.) | 4 | 222446 | Bifidobacterium animalis ssp. Lactis BIFOLAC 12, Bifidobacterium longum BB536, Lactococcus lactis LL-23, Lactobacillus acidophilus LA-14, Lactobacillus rhamnosus BIFOLAC GG, Lactobacillus casei CL-11, Bifidobacterium bifidum BB-02, Lactobacillus delbrueckii ssp. bulgaricus, Lactobacillus plantarum LP-115, Streptococcus thermophilus ST-21; total: 8 mia CFU |
| LACTOCARE FEMININE KAPSLER (30 STK) | 5 | 224327 | Per daily dose (1–2 caps): L. acidophilus (DDS-1) DSMZ 32418: 1–2 mia, L. rhamnosus (GG) DSMZ 32550: 1–2 mia, L. gasseri SD9857: 0.5–1 mia, plus 250–500 mg tranebærekstrakt (18–36 mg PAC), vitamin B2: 0.21–0.42 mg |
| LACTOCARE ADVANCED KAPSLER | 6 | 224334 | L. rhamnosus (GG) DSMZ 32550: 10–20 mia, B. lactis DSMZ 32269: 8.6–3 mia, B. longum DSMZ 32946: 0.14–0.28 mia, B. bifidum DSMZ 32403: 0.14–0.28 mia, L. acidophilus (DDS-1) DSMZ 32418: 0.14–0.28 mia, Lactococcus lactis SD5584: 0.14–0.28 mia, L. casei DSMZ 32382: 0.14–0.28 mia, B. breve SD5206: 0.14–0.28 mia, S. thermophilus DSMZ 32319: 0.14–0.28 mia, L. plantarum DSMZ 32383: 0.14–0.28 mia, L. paracasei DSMZ 26356: 0.14–0.28 mia, L. salivarius SD5208: 0.14–0.28 mia; total: 20–40 mia CFU |
| ALFLOREX KAPSLER | 7 | 225788 | Bifidobacterium longum 35624 |
| LACTOCARE DAILY KAPSLER (30 STK.) | 8 | 224328 | L. rhamnosus (GG) DSMZ 32550, L. acidophilus (DDS-1) DSMZ 32418, zinc citrate, B. longum DSMZ 32946, B. lactis DSMZ 32269, B. bifidum DSMZ 32403, B. breve SD5206, S. thermophilus DSMZ 32319, L. plantarum SD5209 |
| LACTOCARE PLUS KAPSLER | 9 | 224325 | B. lactis DSMZ 32269: 6 mia, L. rhamnosus (GG) DSMZ 32550: 2.5 mia, L. acidophilus (DDS-1) DSMZ 32418: 1 mia, L. paracasei SD5275: 0.5 mia, vitamin B2: 0.21 mg |
| DUOLAC DAGLIG+ VITALITET (60 STK) | 10 | 220800 | S. thermophilus CBT ST3: 1.23 mia, L. acidophilus CBT LA1: 1.23 mia, B. lactis CBT BL3: 1.23 mia, L. rhamnosus CBT LR5: 1.12 mia, B. longum CBT BG7: 1.12 mia, B. bifidum CBT BF3: 1.07 mia; total: 7.0 mia CFU |
| DUOLAC 50+ VITALITET KAPSLER | 11 | 222226 | S. thermophilus CBT ST3: 1.00 mia, L. acidophilus CBT LA1: 1.00 mia, B. lactis CBT BL3: 3.00 mia, L. rhamnosus CBT LR5: 2.00 mia, B. longum CBT BG7: 1.50 mia |
| DUOLAC MIKROBIOM+ | 12 | 222227 | S. thermophilus CBT ST3: 0.34 mia, Pediococcus pentasaceus CBT SL4: 1.90 mia, L. plantarum CBT LP3: 3.10 mia, L. acidophilus CBT LA1: 0.34 mia, L. rhamnosus CBT LR5: 3.10 mia, B. lactis CBT BL3: 3.00 mia, L. casei CBT LC5: 1.90 mia, L. paracasei CBT LPC5: 1.90 mia, B. lactis CBT BL3: 3.10 mia, B. longum CBT BG7: 1.90 mia, B. breve CBT BR3: 1.90 mia, B. bifidum CBT BF3: 0.34 mia, B. infantis CBT BT1: 0.34 mia; total: 20.0 mia CFU; plus vitamins C, B1, B2, B6 |
| GUM PERIOBALANCE SUGETABLETTER | 13 | 212303 | Lactobacillus reuteri Prodentis |
| PROLACSAN SUGETABLETTER | 14 | 225332 | Lactobacillus brevis CECT 7480, Lactobacillus plantarum CECT 7481 |
| LACTOCARE TRAVEL (30 STK.) | 15 | 224321 | Per daily dose (1–2 tablets): Live L. acidophilus: 2.5–5.0 mia; heat-treated L. acidophilus: 2.4–4.8 mia, L. casei: 1.2–2.4 mia, S. thermophilus: 0.4–0.8 mia, plus 0.21–0.42 mg vitamin B6 |
| LACTO SEVEN MÆLKESYRE + INULIN | 16 | 213194 | Per daily dose (1–2 tablets): 1–2 mia CFU total (L. acidophilus, L. casei, L. plantarum, L. reuteri, L. rhamnosus, B. longum, S. thermophilus) plus inulin 300–600 mg |
| LACTO SEVEN KIDS (50 STK) | 17 | 220719 | Per daily dose (1 chewable): 1 mia CFU total (L. acidophilus, L. casei, L. plantarum, L. reuteri, L. rhamnosus, B. longum, S. thermophilus) plus 2 µg vitamin D |
| DUOLAC STOP+ GO 3 I 1 | 19 | 225469 | Per daily dose (2 caps): S. thermophilus CBT ST3: 1.32 mia, L. acidophilus CBT LA1: 1.32 mia, L. plantarum CBT LP3: 2.05 mia, B. lactis CBT BL3: 1.32 mia, total: 6 mia CFU, plus Prolac-T 350 mg, magnesium 56 mg |
| MULTI-TABS TRAVEL PACK | 20 | 225548 | Lacticaseibacillus rhamnosus (LGG), Streptococcus thermophilus STY-31, Lactobacillus acidophilus (LA-5), Lactobacillus bulgaricus LBY-27, Bifidobacterium lactis (BB-12) |
| DUOLAC DAGLIG BØRN+ D | 23 | 226981 | S. thermophilus CBT ST3: 1.70 mia, L. plantarum CBT LP3: 0.56 mia, L. acidophilus CBT LA1: 0.56 mia, L. rhamnosus CBT LR5: 0.56 mia, B. longum CBT BG7: 0.83 mia, B. lactis CBT BL3: 0.83 mia; total: 5.04 mia CFU |
| BIO-KULT BOOSTED KAPSLER | 24 | 224148 | Bacillus subtilis PXN 21, Bifidobacterium bifidum PXN 23, B. breve PXN 25, B. infantis PXN 27, B. longum PXN 30, L. acidophilus PXN 35, L. delbrueckii ssp. bulgaricus PXN 39, L. casei PXN 37, L. plantarum PXN 47, L. rhamnosus PXN 54, L. helveticus PXN 45, L. salivarius PXN 57, Lactococcus lactis ssp. lactis PXN 63, S. thermophilus PXN 66, plus cellulose, HPMC, methylcobalamin |
| RESTOREVÄRN KAPSLER | 25 | 223143 | Per daily dose (1 cap): L. acidophilus NCFM: 2.5 mia, B. lactis Bi-07: 2.5 mia, B. lactis Bi-04: 2.5 mia, L. paracasei Lpc-37: 2.5 mia |
| LACTOCARE DAILY 50+ | 26 | 226317 | L. rhamnosus DSMZ 32550, L. plantarum SD520, B. lactis DSMZ 32269, B. breve SD520, B. longum DSMZ 32946, B. bifidum DSMZ 32403, zinc citrate, vitamin D3, vitamin B1 |
| BIOSYM SYMBIOFLOR+ (60 STK.) | 27 | 218988 | L. acidophilus La-14: 3.75 mia, B. lactis Bi-07: 1.0 mia, L. rhamnosus Lr-32: 0.25 mia, total: 5.0 mia CFU |
| LACTOCARE REBALANCE | 28 | 224323 | Per daily dose (2 caps): heat-treated L. acidophilus LMG S-24802: 4.8 mia, L. casei LMG S-24115: 2.4 mia, S. thermophilus LMG S-24116: 0.8 mia, total 8 mia, plus Saccharomyces cerevisiae var. boulardii CNCM I-3799: 5.0 mia |
| LACTOCARE DIGESTIVE COMFORT | 29 | 225229 | L. acidophilus (DDS-1) DSMZ 32418: 0.3 mia, L. rhamnosus GG DSMZ 32550: 0.9 mia, B. lactis DSMZ 32269: 1.2 mia, B. longum DSMZ 32946: 0.3 mia, B. bifidum DSMZ 32403: 0.3 mia |
| SENIORVÄRN KAPSLER | 30 | 220695 | B. lactis HN019: 7 mia, B. bifidum: 1 mia, L. acidophilus: 1 mia, L. plantarum: 1 mia |
| LACTOCARE STOP KAPSLER | 31 | 224322 | Per daily dose (2 caps): S. cerevisiae var. boulardii CNCM I-3799: 5.0 mia, heat-treated L. acidophilus LMG S-24802: 4.8 mia, L. casei LMG S-24115: 2.4 mia, S. thermophilus LMG S-24116: 0.8 mia, plus magnesium 56.25 mg |
| ADULTVÄRN KAPSLER | 32 | 223497 | B. bifidum Bb-06: 2 mia, B. lactis BI-04: 2 mia, B. lactis HN019: 2 mia, L. acidophilus La-14: 2 mia, L. brevis Lbr-35: 1 mia, L. casei Lc-11: 2 mia, L. paracasei Lpc-37: 1 mia, Lactococcus lactis LI-23: 2 mia, S. thermophilus St-21: 1 mia |
| FEMIDUR KAPSLER (10 STK) | 33 | 214200 | L. rhamnosus GR-1, L. reuteri RC-14: 5 mia |
| LACTOCARE PREGNANT KAPSLER | 34 | 224324 | B. lactis DSMZ 32269, L. reuteri CBS 145621, L. rhamnosus DSMZ 26357, plus fructooligosaccharider, m.m. |
| Lacto lady | 35 | 213196 | L. acidophilus: 2.5 x 10 mia, L. casei: 2.5 x 10 mia, L. rhamnosus: 2.5 x 10 mia, B. longum: 2.5 x 10 mia |
| SACCHAFLOR KAPSLER | 36 | 210335 | S. boulardii (DBVPG 6763) |
