# Supplementary material for: Multi-omics analysis of saccharomyces boulardii supplementation reveals coordinated microbiome, metabolic, and immune signaling changes accompanying tumor suppression
Source: Gut Microbes. 2026 Jun 30;18(1):2690687. doi: 10.1080/19490976.2026.2690687 (PMC13336259; doi:10.1080/19490976.2026.2690687)
Supplement: Supplemental Material — Supplementary Table 2 [file KGMI_A_2690687_SM5729.docx]

Table 2– KEGG modules used to assess metabolic independence score of metagenome assembled genomes

| **Module** | **Module name** | **Reaction** | **Pathway class** |
| --- | --- | --- | --- |
| M00049 | Adenine ribonucleotide biosynthesis | IMP → ADP, ATP | Nucleotide metabolism → Purine metabolism |
| M00050 | Guanine ribonucleotide biosynthesis | IMP → GDP, GTP | Nucleotide metabolism → Purine metabolism |
| M00007 | Pentose phosphate pathway, non‑oxidative phase | fructose 6P → ribose 5P | Carbohydrate metabolism → Central carbohydrate metabolism |
| M00140 | C1‑unit interconversion, prokaryotes | — | Metabolism of cofactors and vitamins → Cofactor and vitamin metabolism |
| M00005 | PRPP biosynthesis | ribose 5P → PRPP | Carbohydrate metabolism → Central carbohydrate metabolism |
| M00083 | Fatty acid biosynthesis, elongation | — | Lipid metabolism → Fatty acid metabolism |
| M00120 | Coenzyme A biosynthesis | pantothenate → CoA | Metabolism of cofactors and vitamins |
| M00854 | Glycogen biosynthesis | glucose‑1P → glycogen/starch | Carbohydrate metabolism |
| M00527 | Lysine biosynthesis, DAP aminotransferase pathway | aspartate → lysine | Amino acid metabolism |
| M00096 | C5 isoprenoid biosynthesis, non‑mevalonate pathway | — | Biosynthesis of terpenoids and polyketides |
| M00048 | Inosine monophosphate biosynthesis | PRPP + glutamine → IMP | Nucleotide metabolism |
| M00855 | Glycogen degradation | glycogen → glucose‑6P | Carbohydrate metabolism |
| M00022 | Shikimate pathway | phosphoenolpyruvate + erythrose‑4P → chorismate | Amino acid metabolism |
| M00844 | Arginine biosynthesis | ornithine → arginine | Amino acid metabolism |
| M00051 | Uridine monophosphate biosynthesis | glutamine (+ PRPP) → UMP | Nucleotide metabolism |
| M00082 | Fatty acid biosynthesis, initiation | — | Lipid metabolism |
| M00157 | F‑type ATPase, prokaryotes and chloroplasts | — | Energy metabolism |
| M00026 | Histidine biosynthesis | PRPP → histidine | Amino acid metabolism |
| M00526 | Lysine biosynthesis, DAP dehydrogenase pathway | aspartate → lysine | Amino acid metabolism |
| M00015 | Proline biosynthesis | glutamate → proline | Amino acid metabolism |
| M00019 | Valine/isoleucine biosynthesis | pyruvate → valine / 2‑oxobutanoate → isoleucine | Amino acid metabolism |
| M00432 | Leucine biosynthesis | 2‑oxoisovalerate → 2‑oxoisocaproate | Amino acid metabolism |
| M00018 | Threonine biosynthesis | aspartate → homoserine → threonine | Amino acid metabolism |
| M00570 | Isoleucine biosynthesis | threonine → 2‑oxobutanoate → isoleucine | Amino acid metabolism |
| M00126 | Tetrahydrofolate (THF) biosynthesis | GTP → THF | Metabolism of cofactors and vitamins |
| M00115 | NAD biosynthesis | aspartate → quinolinate → NAD | Metabolism of cofactors and vitamins |
| M00028 | Ornithine biosynthesis | glutamate → ornithine | Amino acid metabolism |
| M00924 | Cobalamin biosynthesis (anaerobic, early steps) | uroporphyrinogen III → sirohydrochlorin → cobyrinate a,c‑diamide | Metabolism of cofactors and vitamins |
| M00122 | Cobalamin biosynthesis (late steps) | cobyrinate a,c‑diamide → cobalamin | Metabolism of cofactors and vitamins |
| M00125 | Riboflavin biosynthesis (plants & bacteria) | GTP → riboflavin/FMN/FAD | Metabolism of cofactors and vitamins |
| M00023 | Tryptophan biosynthesis | chorismate → tryptophan | Amino acid metabolism |
| M00631 | D‑Galacturonate degradation (bacteria) | D‑galacturonate → pyruvate + D‑glyceraldehyde‑3P | Carbohydrate metabolism |
| M00061 | D‑Glucuronate degradation | D‑glucuronate → pyruvate + D‑glyceraldehyde‑3P | Carbohydrate metabolism |
