# Supplementary material for: Multi-omics analysis of saccharomyces boulardii supplementation reveals coordinated microbiome, metabolic, and immune signaling changes accompanying tumor suppression
Source: Gut Microbes. 2026 Jun 30;18(1):2690687. doi: 10.1080/19490976.2026.2690687 (PMC13336259; doi:10.1080/19490976.2026.2690687)
Supplement: Supplementary_figure.docx — Supplemental Material [file KGMI_A_2690687_SM7403.docx]

## Appendices

## Supplementary Figures


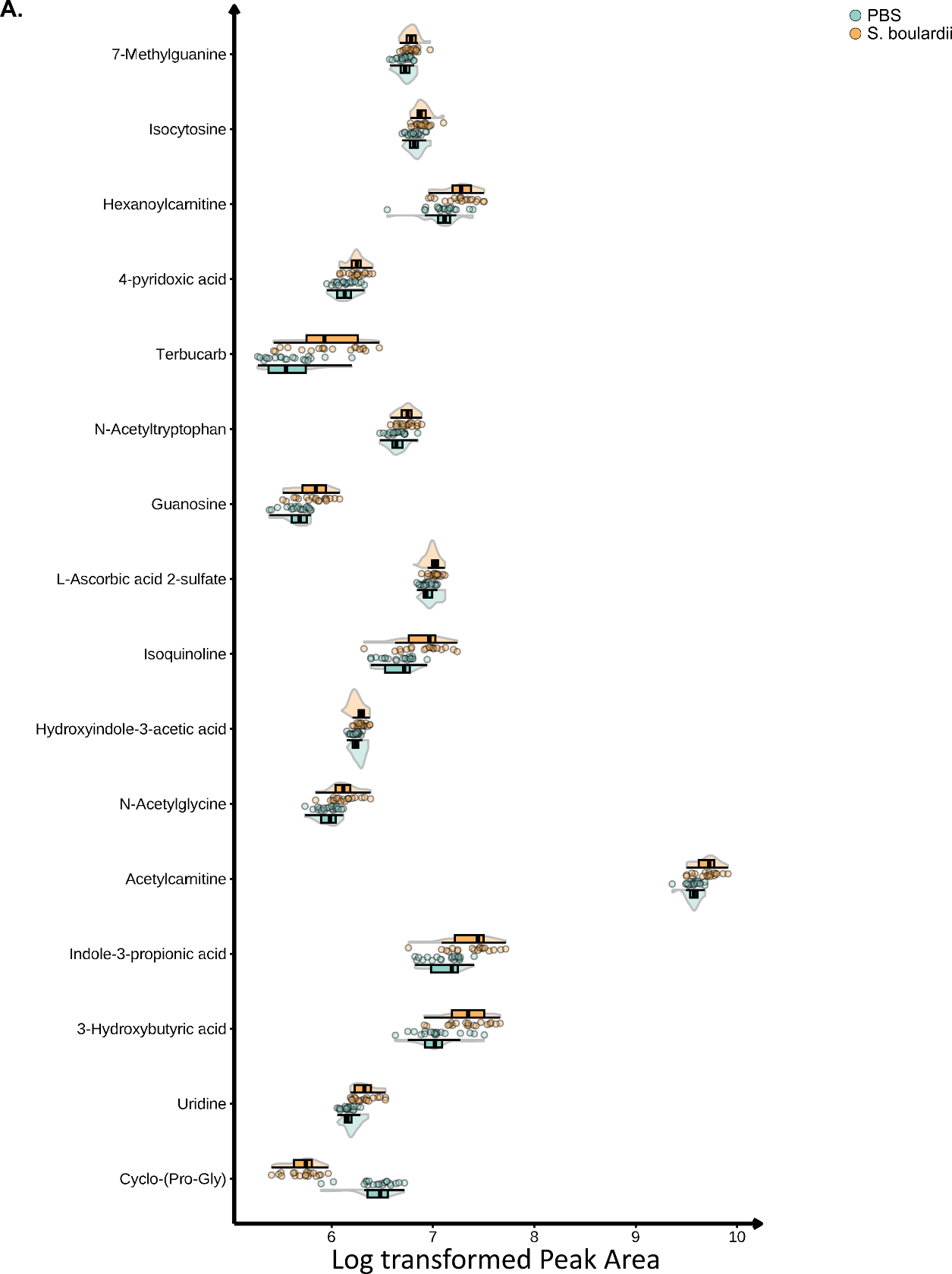


**Figure S1 – Significantly altered plasma metabolites following S. boulardii supplementation.**
Raindrop plots showing the 16 metabolites significantly different between S. boulardii–treated and PBS control mice, as identified by untargeted plasma metabolomics. Peak areas were log_10_-transformed and are shown for each individual mouse (n ≥ 19 per group). Data are grouped by treatment, with distributions visualized as half-violin plots overlaid with boxplots and individual data points.


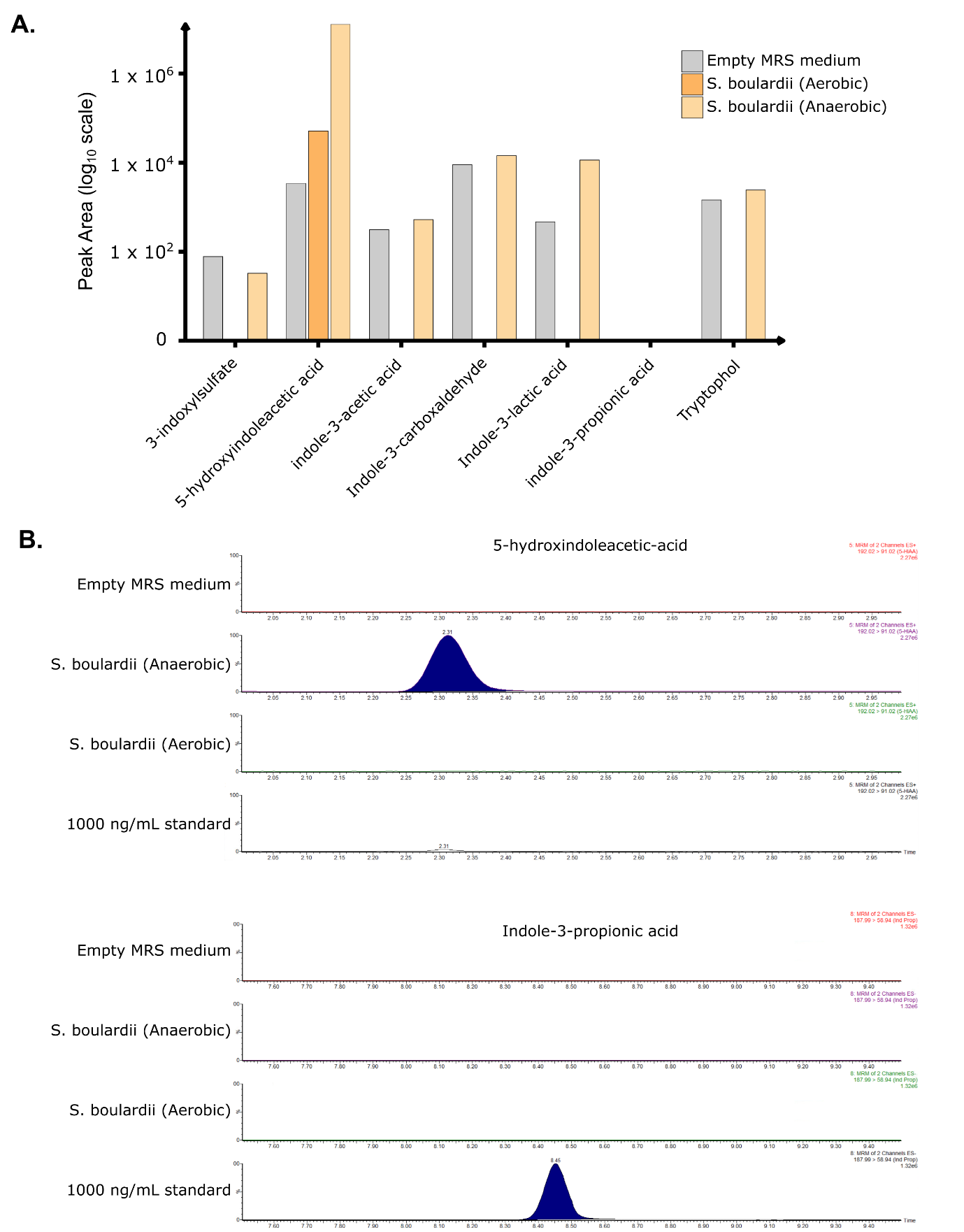


**Figure S2 – Targeted LC–MS/MS analysis of indole derivatives in S. boulardii spent medium**
**(A)** Spent medium from S. boulardii cultures grown in MRS broth under aerobic and anaerobic conditions incubated at 37°C for 48 hours under static conditions, alongside sterile MRS controls, were analyzed by targeted UPLC–MS/MS. The targeted panel included 5-hydroxyindole-3-acetic acid (5-HIAA), indole-3-acetic acid (IAA), indole-3-carboxaldehyde, indole-3-lactic acid (ILA), indole-3-propionic acid (IPA), 3-indoxylsulfate, tryptophol (calibration standards = 1,000 ng/mL). **(B)** Extracted ion chromatograms for 5-hydroxyindole-3-acetic acid (5-HIAA) and indole-3-propionic acid (IPA) from the same samples shown in panel A, together with a 1000 ng/mL calibration standard.


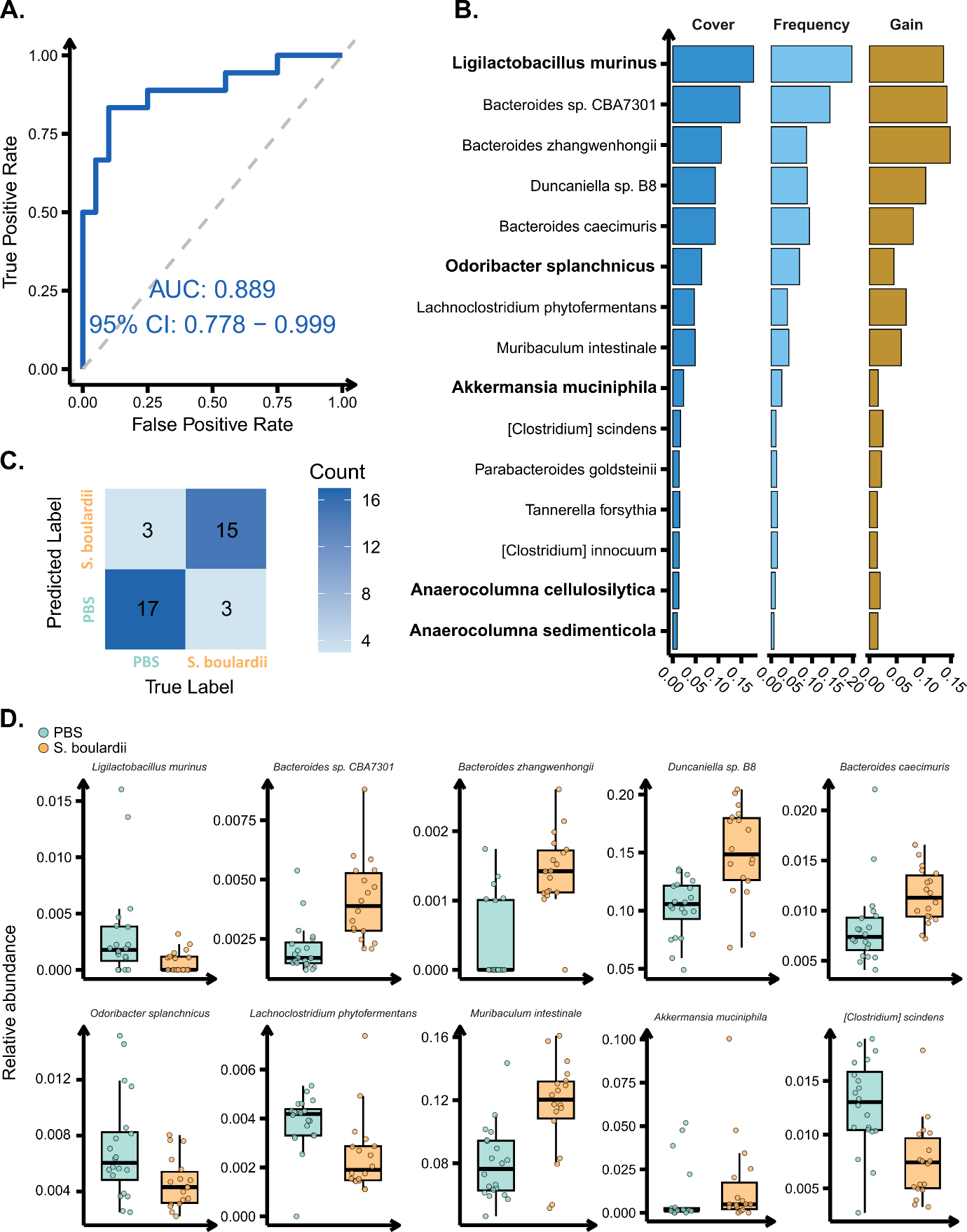


**Figure S3** **– Machine learning classification of microbiome profiles accurately separates S. boulardii–treated and control mice.**
**(A)** Receiver operating characteristic (ROC) curve showing performance of an extreme gradient boosting (XGBoost) classifier trained on species-level microbial relative abundance profiles to distinguish S. boulardii and PBS-treated mice. The model achieved a cross-validated area under the curve (AUC) of 0.889 (95% CI: 0.778–0.999). **(B)** Feature importance scores for the top 15 taxa contributing to classification performance, shown across three XGBoost-derived metrics: Cover (proportion of samples affected), Frequency (times used in splits), and Gain (improvement in accuracy). **(C)** Confusion matrix showing classification performance on the validation set. **(D)** Relative abundance of the top 10 predictive species, stratified by treatment group.


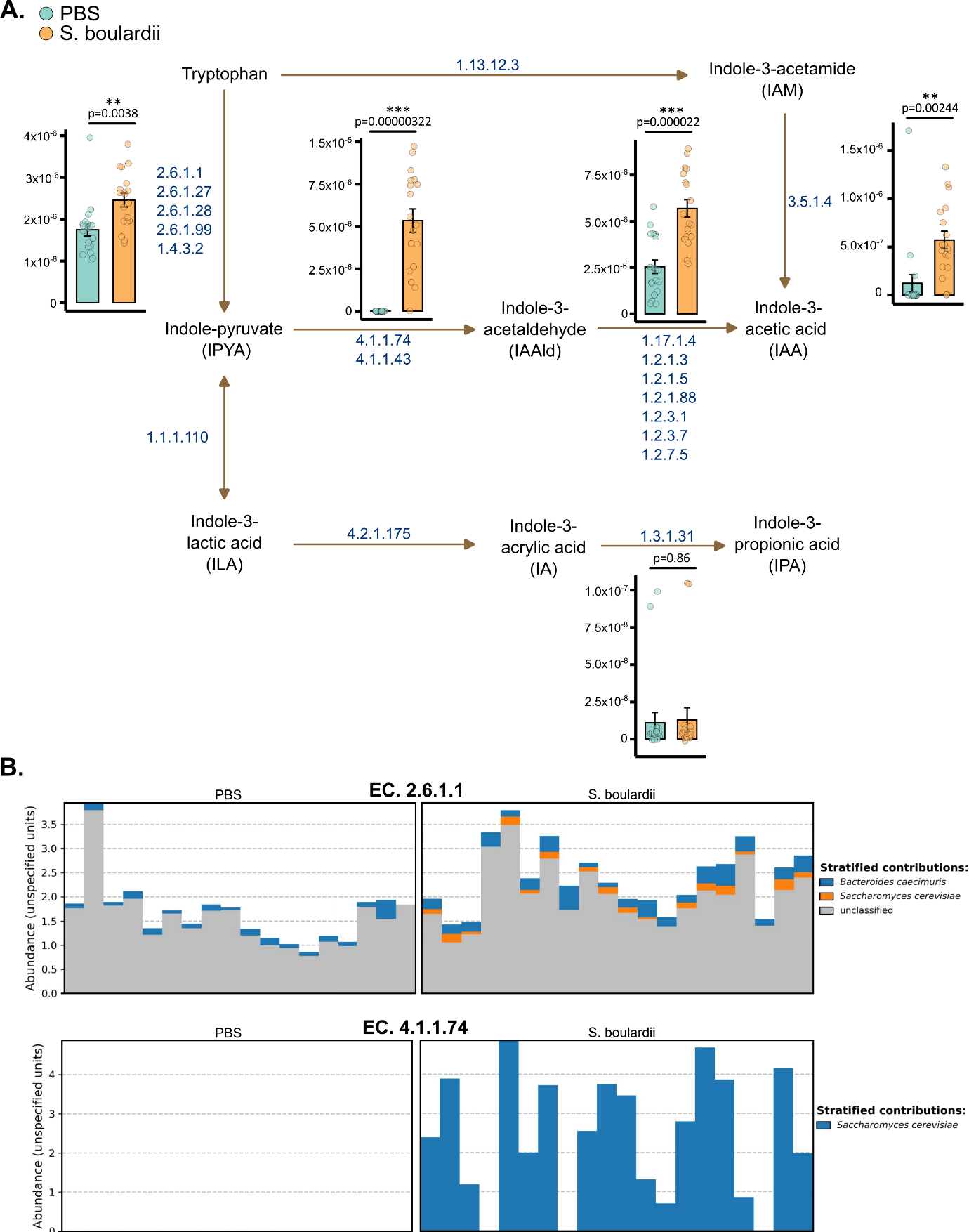


**Figure S4 – Microbial pathway analysis of indole derivative biosynthesis following S. boulardii treatment.**
**(A)** Bar plots showing the relative abundance of microbial genes encoding enzymes involved in tryptophan-derived indole metabolism. Enzymatic reactions are annotated with Enzyme Commission (EC) numbers and are arranged to reflect the biosynthetic pathway from tryptophan to various indole derivatives, including indole-3-lactic acid (ILA), indole-3-acetaldehyde (IAALd), indole-3-acetamide (IAM), indole-3-acetic acid (IAA), indole-3-acrylic acid (IA), and indole-3-propionic acid (IPA). Each bar represents mean ± SEM, with individual mouse-level abundances shown as dots. Group differences were evaluated using two-sided Welch’s t-tests, and multiple comparisons across all EC features were corrected using the Benjamini–Hochberg false discovery rate (FDR), *p<0.05, **p<0.01, ***p<0.001. **(B)** Stratified HUMAnN3 output showing taxon-stratified contributions to the abundances of EC 2.6.1.1 and EC 4.1.1.74 enzymes in PBS and S. boulardii–treated groups. Each bar represents the total abundance of the given EC feature per sample, and the segments within each bar show the stratified contributions of individual taxa to that feature.


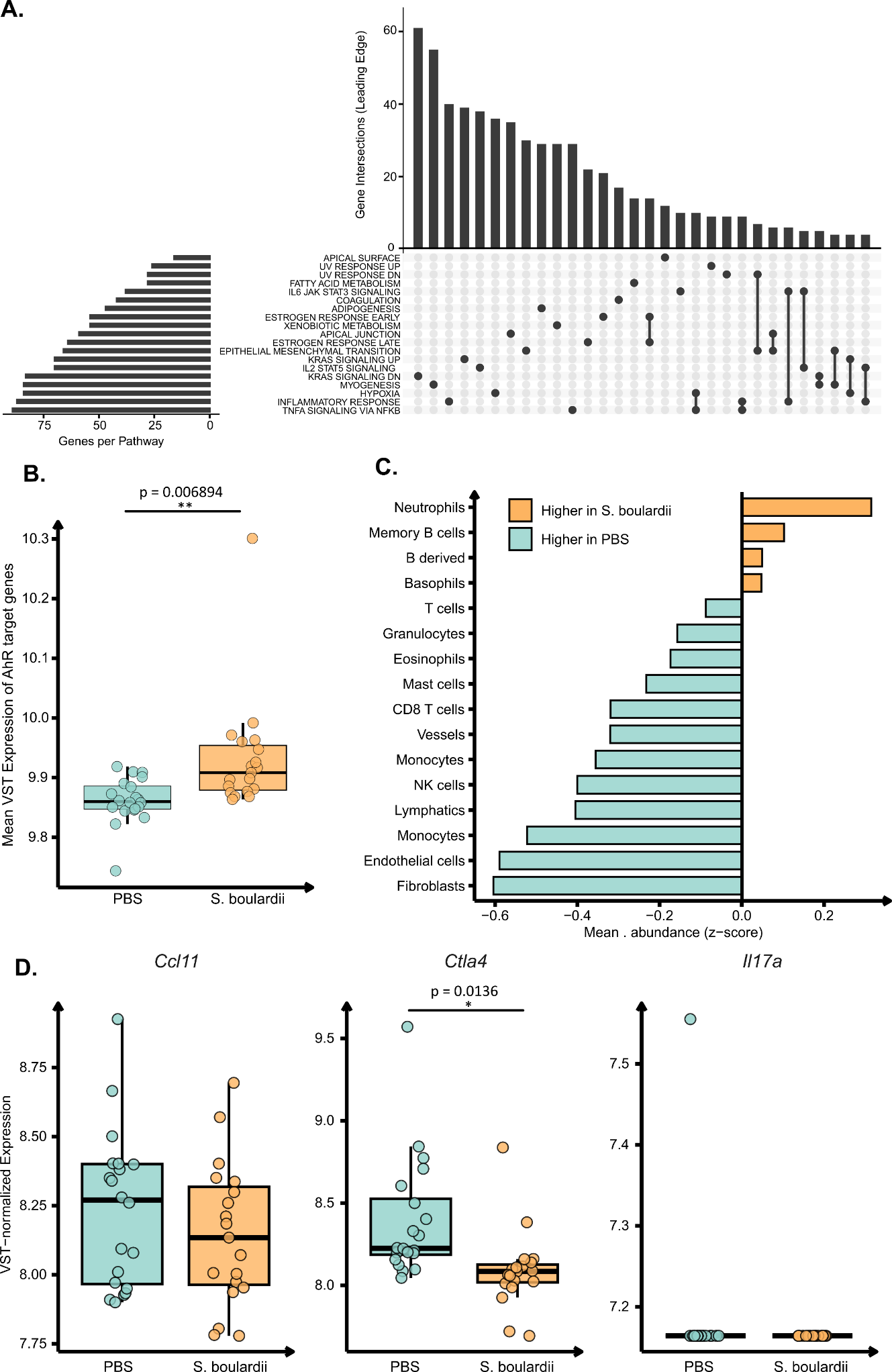


**Figure S5 – Transcriptomic analyses of tumor RNA-seq data.**
**(A)** UpSet plot showing gene overlap across the leading-edge subsets of significant pathways. Top bar plot displays the number of shared genes between combinations of leading-edge gene sets, while the left bar plot shows the total number of genes per individual pathway. **(B)** Mean variance-stabilized expression (VST) values for genes annotated as AhR transcription factor targets. The AhR target set was obtained from the ChEA database and comprised 666 genes, of which 615 could be mapped to mouse gene identifiers; 581 genes with non-zero expression were included in the analysis. Each point represents an individual tumor sample (Welch’s two-sample t-test, p = 0.0069). **(C)** Inferred immune- and stromal-cell abundances estimated from bulk tumor RNA-seq using mMCP-counter. Bars show the mean z-score–normalized abundance for each cell population in each treatment group (no significant differences detected across cell types after FDR correction; global composition test by PERMANOVA: R² = 0.03, p = 0.322). **(D)** Validation of genes highlighted by plasma proteomics in Figure 2A. Boxplots show VST-normalized expression of Ccl11, Ctla4, and Il17a in MC38 tumors from PBS- and S. boulardii–treated mice; dots represent individual animals and boxes indicate median with interquartile range. Group differences were evaluated using two-sided Welch’s t-tests with Benjamini–Hochberg FDR correction across the three genes.

Table 3 – High quality metagenome assembled genomes and their metabolic independence score
